# Competition in Notch Signaling with Cis Enriches Cell Fate Decisions

**DOI:** 10.1371/journal.pone.0095744

**Published:** 2014-04-29

**Authors:** Pau Formosa-Jordan, Marta Ibañes

**Affiliations:** Dept. Estructura i Constituents de la Matèria, Facultat de Física, Universitat de Barcelona, Barcelona, Spain; Universitat Pompeu Fabra, Spain

## Abstract

Notch signaling is involved in cell fate choices during the embryonic development of Metazoa. Commonly, Notch signaling arises from the binding of the Notch receptor to its ligands in adjacent cells driving cell-to-cell communication. Yet, cell-autonomous control of Notch signaling through both ligand-dependent and ligand-independent mechanisms is known to occur as well. Examples include Notch signaling arising in the absence of ligand binding, and cis-inhibition of Notch signaling by titration of the Notch receptor upon binding to its ligands within a single cell. Increasing experimental evidences support that the binding of the Notch receptor with its ligands within a cell (cis-interactions) can also trigger a cell-autonomous Notch signal (cis-signaling), whose potential effects on cell fate decisions and patterning remain poorly understood. To address this question, herein we mathematically and computationally investigate the cell states arising from the combination of cis-signaling with additional Notch signaling sources, which are either cell-autonomous or involve cell-to-cell communication. Our study shows that cis-signaling can switch from driving cis-activation to effectively perform cis-inhibition and identifies under which conditions this switch occurs. This switch relies on the competition between Notch signaling sources, which share the same receptor but differ in their signaling efficiency. We propose that the role of cis-interactions and their signaling on fine-grained patterning and cell fate decisions is dependent on whether they drive cis-inhibition or cis-activation, which could be controlled during development. Specifically, cis-inhibition and not cis-activation facilitates patterning and enriches it by modulating the ratio of cells in the high-ligand expression state, by enabling additional periodic patterns like stripes and by allowing localized patterning highly sensitive to the precursor state and cell-autonomous bistability. Our study exemplifies the complexity of regulations when multiple signaling sources share the same receptor and provides the tools for their characterization.

## Introduction

The Notch signaling pathway mediates cell-to-cell communication in several developmental contexts [1]–[4]. This communication occurs through the binding of the Notch receptor in a cell membrane to its ligand (*e.g*. Delta) in a neighboring cell [5], what herein we refer as trans-interactions. The bound complex is then cleaved and its intracellular domain (NICD) targets gene expression within the cell that harbors the bound receptor [6]. In the case of neural development, Notch signaling mediates lateral inhibition, which drives the selection of cells that express high levels of proneural genes and ultimately become neurons [7]–[11]. Notch signaling activity inhibits the proneural genes, which in turn activate Delta expression. As a result, a cell committed to the neural fate with high proneural gene and Delta levels inhibits its neighboring cells from adopting the same fate (*i.e*. it performs lateral inhibition) [12]. When all precursor cells are initially equivalent and signal similarly, mutual lateral inhibition arises and can drive a spontaneous spatially periodic selection of precursors by amplifying the initial small differences between them [13].

Different experimental evidences show that the Notch receptor can bind as well to the ligands when they are both in the same cell, what we refer as cis-interactions [5], [14]–[18]. Cis-interactions drive a reduction of Notch signaling by sequestering the receptor and impeding its signaling [14], [16], [18]–[28]. This is known as cis-inhibition and its effects have started to be theoretically and computationally addressed too [25], [29]–[38]. These studies have revealed that cis-inhibition can facilitate patterning by promoting faster responses, enhancing robustness and precision, and relaxing the constraints required for patterning [25], [29], [32], [33], [35], [37], [38].

Although cis-interactions commonly inhibit Notch signaling [14], [16], [18]–[28], Notch activity coming from cis-interactions has been proposed for specific scenarios [39]–[43]. For instance, Coumailleau *et al*. (2009) pointed to cell-autonomous Delta-dependent active Notch in Sara endosomes of *Drosophila* bristle precursor cells. Guy *et al*. (2013) indicated that cell-autonomous Notch-mediated activation of the cell-cycle regulator c-Myc in mouse T-cells is impaired when ligand-receptor cis-binding is prevented. The mechanism by which cis-driven signaling can occur in these scenarios is still missing. According to the mechanism proposed by Fürthauer and González-Gaitán (2009), ligand-receptor binding within multivesicular endosomes could drive the release of Notch intracellular domain, driving Notch activation. This binding would occur in anti-parallel configurations (like for trans-interactions), as opposed to the parallel binding that is commonly associated to cis-interactions when occurring in the cell membrane and which is believed to prevent signaling. The interplay between Notch and the endocytic routes is starting to be uncovered and may shed light on this issue.

**Figure 1:**
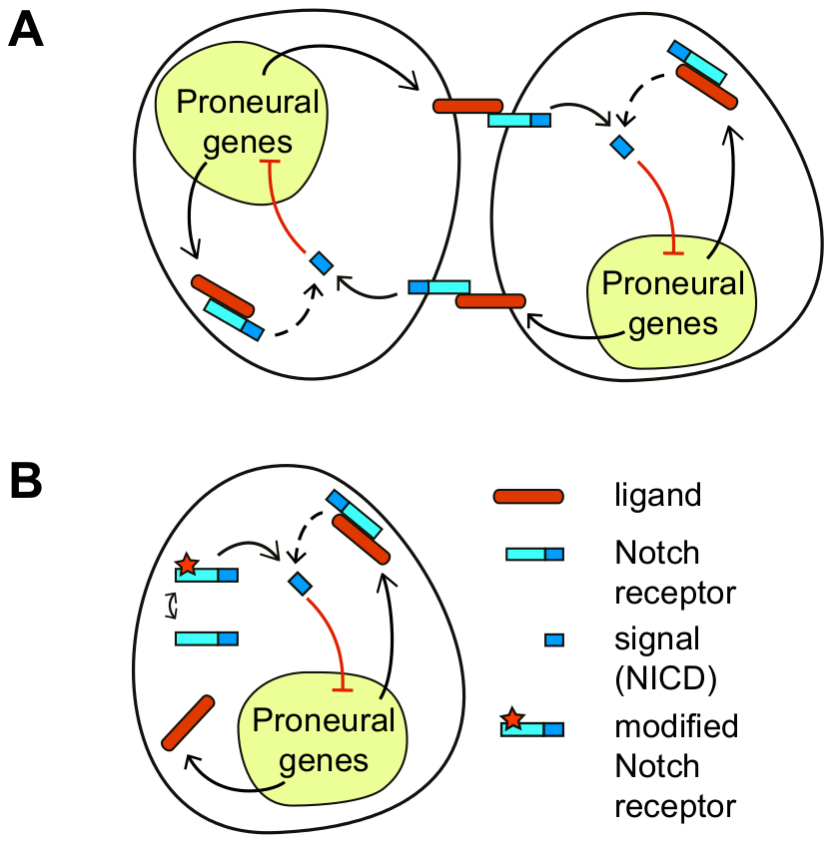
A model for Notch signaling driven by a primary signaling source and by cis-interactions. Cartoons of the Notch signaling components under study for (A) two adjacent cells that interact and for (B) an isolated cell. Black arrows stand for activation while red blunt arrows denote inhibition. (A) The ligand (red) in a cell binds the Notch receptor (blue) in a neighboring adjacent cell (trans-interactions). This elicits a Notch signal (NICD) that inhibits ligand production in the adjacent cell. The ligand can also bind the receptor within the same cell (cis-interactions) and drive Notch signaling at a different strength (dashed arrow). (B) A primary signaling source that is ligand-independent is depicted as well as signaling driven by cis-interactions. In both panels, the Notch signal inhibits the ligand through the proneural genes.

In the present work we take advantage of computational and mathematical modeling to address the question of which would be the effect expected from cis-signaling when another signaling source that also uses the Notch receptor is acting (Fig. 1). Since both signaling sources use the Notch receptor, both can compete for it. The additional source of signaling, hereinafter referred to as primary signaling source, can be associated with trans-interactions. Yet, our modeling approach is not exclusive for such trans-interactions. The primary signal can be also driven by additional alternative mechanisms. In this context, recent work has shown that ligand-independent Notch activity can arise from the binding of Notch to other factors and from impaired endocytic regulation [39], [40], [44]–[54]. For instance, a recent study in *Drosophila* blood cells has detected a ligand-independent Notch signal that has a significant role in their development [52], [53]. Our results show that when acting together with a primary signal, cis-signaling can act as cis-activation or as cis-inhibition. Competition between signaling sources underlies this switch. We establish under which conditions each regime arises. An extensive analysis of the parameter space shows that cis-inhibition enriches patterning, as opposed to cis-activation. Cis-inhibition promotes pattern multistability and modulates the selection of precursors. In addition, cis-inhibition facilitates cell-autonomous bistability.

## Results

### A simple model for lateral inhibition with cis-signaling

We set a mathematical phenomenological model that includes two sources of Notch signaling: a primary signaling source and signaling driven by cis-interactions between the Notch receptor and its ligand within the same cell. The primary signal can be driven either by trans-interactions between the Notch receptor and its ligand in an adjacent cell (Fig. 1A) or through ligand-independent mechanisms (Fig. 1B). We assumed that both the primary signaling source and cis-interactions drive the same type of signal. We set primary Notch signaling to occur in a graded non switch-like fashion and to saturate to a maximal value, as recently experimentally reported for trans-interactions [25]. We assumed that cis-interactions can drive a graded increasing production of Notch signal activity with ligand up to saturation like the primary signaling source does (Methods). When both signaling sources are acting, the production of signal that each of them drives depends on the other source since both sources use the Notch receptor to signal. Accordingly, these productions in cell 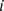 read (Methods):
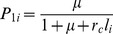
(1)




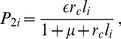
(2)where the primary source produces Notch signal activity at rate 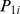 whereas cis-interactions produce it at rate 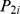. In the above equations 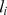 stands for the ligand activity in cell 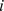 and 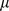 is a measure of the amount of primary signaling source. Non-dimensional units are used with maximal signal production being 1 for the primary signal without any loss of generality.

We considered that each source can drive Notch activity production at a different rate such that it results into different values of the stationary saturated Notch activity. Parameter 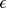 accounts for the ratio of the stationary saturated Notch activity driven by cis-interactions over that one driven by the primary signaling source. A more complex biochemical reaction-based scheme indicates that 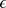 can be understood as the ratio between the signaling efficiency of the two sources, which depends on the signaling rate and the stability of each source (Methods). Therefore, we refer to 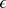 as relative signaling efficiency. 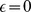 corresponds to the well-known cis-inhibition, in which cis-interactions titrate the receptor and drive no signaling. When 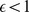, cis-interactions drive signaling less efficiently than the primary signaling source.

When the primary signaling source is driven by trans-interactions we have 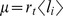, where 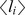 stands for the weighted average of ligand activity in cells adjacent to cell 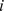 (Methods). Parameters 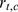 arising for each Notch signaling source are the trans and cis-interactions strengths, which are related to the trans and cis-binding and unbinding rates of the receptor and ligand complexes (see Methods for their definition in a more biochemical reaction-based approach). The trans and cis-interactions strengths parameters 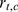 set the threshold values of ligand for signal activation.

We set production of ligand to be inhibited by Notch signaling and took linear degradation for both signal and ligand activities (Methods). Taken together, the dynamics in cell 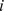 of the Notch signal 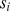 and the ligand 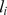 activities read:
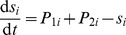
(3)




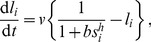
(4)where time 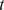 is non-dimensional, 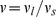, being 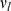 and 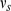 the degradation rates of the ligand and Notch signal respectively, 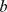 stands for the strength of ligand inhibition through Notch signaling and 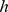 represents an effective cooperativity of such an inhibition. When cis-interactions are not present (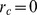), Eqs. 1–4 reduce to the model for Delta/Notch-mediated lateral inhibition dynamics early proposed by Collier *et al*. [13].

### A switch from cis-activation to cis-inhibition

**Figure 2:**
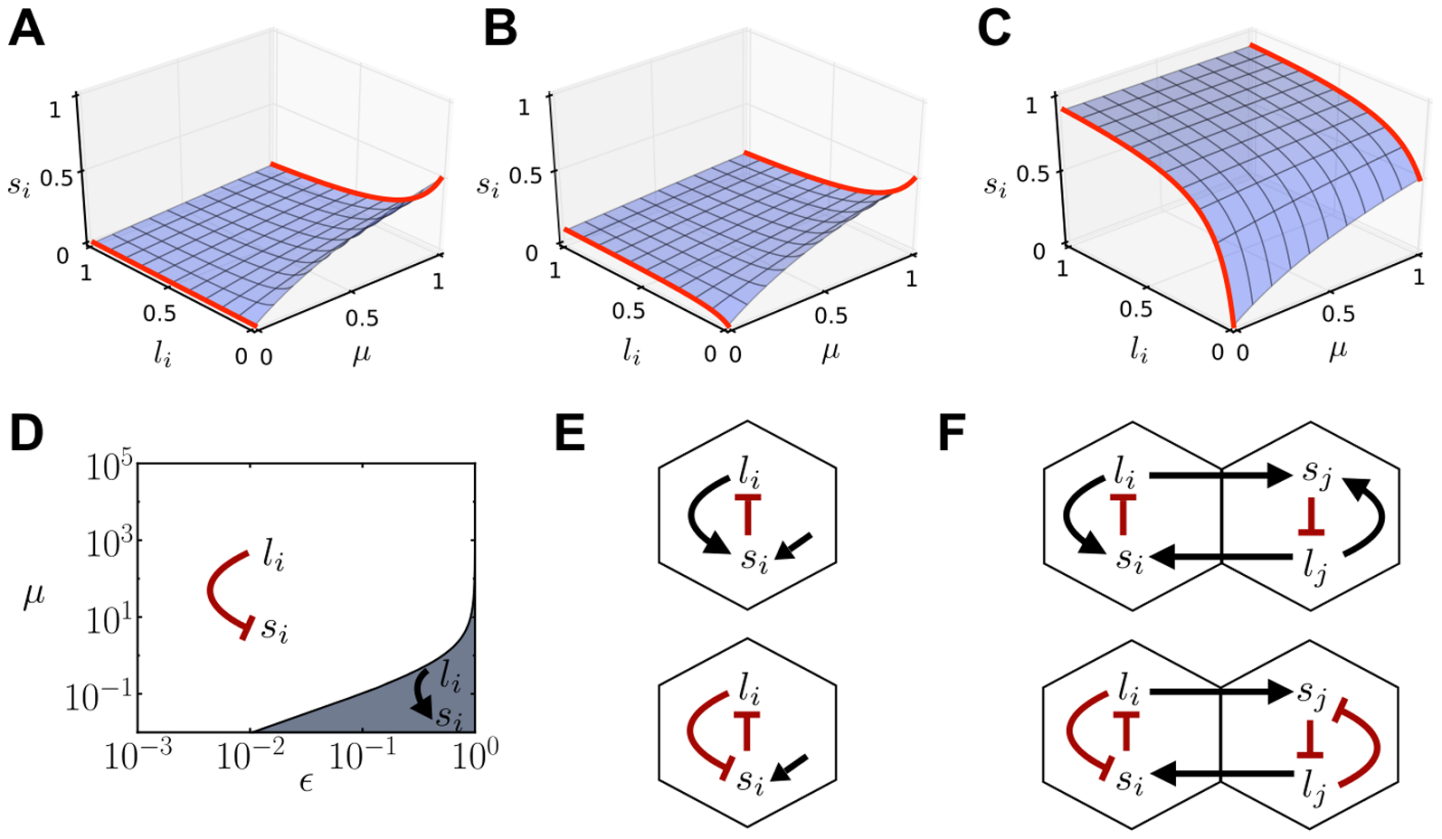
A switch between cis-activation and cis-inhibition. (A–C) Stationary Notch signal in a cell (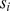) *versus* the amount of ligand in that cell (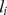) and the amount of primary signaling source (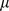) for (A) 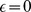, (B) 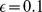 and (C) 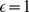. Red lines show the Notch signal dependence on 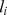 in the absence of the primary signaling source (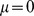) and for a primary signaling source with 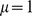. Decreasing curves indicate cis-inhibition and increasing curves show cis-activation. In B, cis-interactions drive cis-activation at low 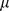 values, whereas they drive cis-inhibition at higher 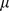. (D) Parameter space showing where cis-activation (gray region) and cis-inhibition (white region) occurs, according to inequality 5. (E–F) Effective circuit architectures of the model when cis-interactions drive cis-activation (top) and cis-inhibition (bottom) for (E) isolated cells with a primary signaling source (straight arrow) and for (F) two adjacent cells that interact through trans-binding. Black arrows stand for activation, while red blunt arrows for inhibition. Parameter values: 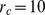 in panels A–C, 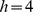 and 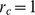 in panel D.

We first evaluated whether the total Notch signaling increases (cis-activation) or decreases (cis-inhibition) when cis-interactions that drive Notch signaling are included. From Eqs. 1–3, it is obtained that cis-inhibition always occurs when cis-interactions do not drive a signal (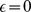) as expected (Fig. 2A). In contrast, either cis-inhibition or cis-activation can arise when cis-interactions drive signaling on their own (Fig. 2B,C).

We explored under which conditions each cis-signaling regulatory role (cis-inhibition *versus* cis-activation) arises and found that cis-inhibition occurs for (Fig. 2D, Methods)
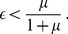
(5)Otherwise, cis-interactions drive cis-activation (Fig. 2D). According to the above relation, cis-inhibition requires cis-signaling to be less efficient than the primary signaling source (*i.e*. 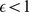). Yet, less efficient cis-signaling does not ensure cis-inhibition. The above relation shows that the regulatory role of cis-signaling depends on the amount of the primary signaling source (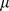) as well. When the primary signaling source comes from trans-interactions, the regulatory role of cis-signaling depends on the trans-interactions strength (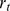). For 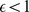, the regulatory role switches from cis-activation to cis-inhibition as the primary source becomes more abundant (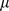 or 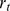 increases) (Fig. 2B).

The above result indicates that whether cis-signaling is acting as cis-activation or cis-inhibition does not depend on the strength of cis-interactions (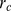; Fig. S1). This is because the qualitative change (decrease or increase) in Notch signaling driven by the addition of ligand within a cell with primary Notch signal activity is independent of the amount of ligand being added. In contrast, the quantitative change depends on 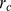 and on the amount of added ligand (Fig. S1C).

### Cis-signaling can drive two distinct effective circuit architectures

The above analysis only took into account the dynamics of the signal when two sources of signaling (primary and cis-driven) are competing for the Notch receptor, Eqs. 1–3. We then asked which is the effect of this switch between cis-inhibition and cis-activation on the overall signal and ligand dynamics. To this end, we first evaluated the effective genetic circuits that arise when the dynamics of the ligand, Eq. 4, is included. By “effective” we mean that the circuit does not describe the individual interactions *per se* but their resulting regulatory role, which takes into account the context in which they occur. Therefore, we considered which circuit architecture arises when cis-interactions perform cis-activation and when they drive cis-inhibition. It can be readily seen that cis-interactions, coupled to a primary signal, give rise to two different effective genetic circuits (Fig. 2E). When cis-interactions drive cis-activation, a negative intracellular transcriptional feedback loop arises. In contrast, a positive feedback loop emerges when cis-interactions drive cis-inhibition. It is worth stressing that the amount of primary signal (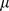) and the relative cis to primary signaling efficiencies (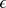) control which of the two effective architectures is acting by setting which is the regulatory role of cis-signaling.

When the primary source is trans-interactions, these intracellular feedbacks are coupled to the intercellular mutual inhibition loop that is characteristic of lateral inhibition (Fig. 2F). Positive and negative feedback loops are well known to drive different dynamics, like bistability for the former and homeostasis for the latter (see for instance [55]). Accordingly, we can expect different roles of cis-signaling on patterning and cell fate choices, depending on whether it is in the cis-inhibition or cis-activation regime.

### Cis-activation inhibits fine-grained pattern formation, while optimal values of cis-inhibition promotes it

**Figure 3:**
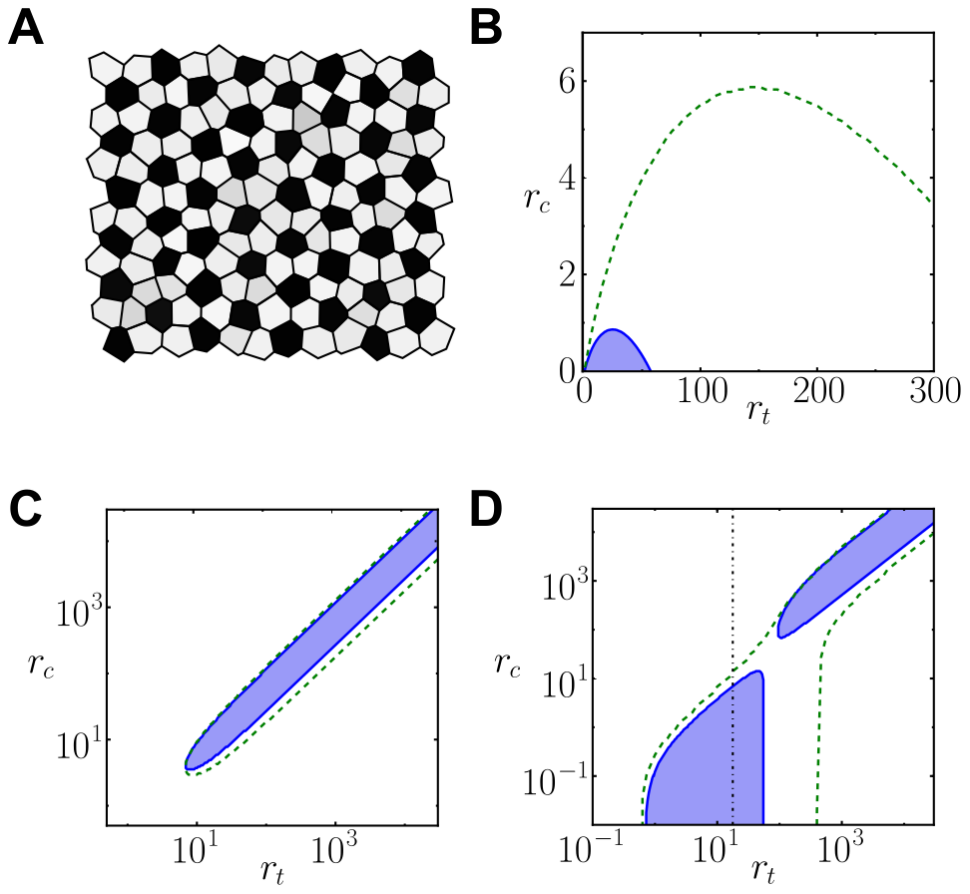
Cis-activation inhibits patterning and cis-inhibition facilitates it. (A) Stationary lateral inhibition pattern formed in an array of irregular cells in the absence of cis-interactions (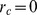). Grayscale is used to denote the ligand level (black for high ligand, 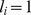, and white for low ligand, 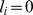). (B–D) Regions of patterning for cis-interactions and trans-interactions strengths 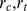 for (B) 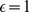 (cis-activation), (C) 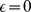 (cis-inhibition) and (D) 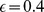. The black dot-dashed line in D divides the parameter space into the cis-activation region, on its left, and the cis-inhibition region, on its right. Blue regions show where the pattern grows spontaneously (LSA in Methods). Green dashed lines enclose the regions where lateral inhibition pattern solutions exist and are stable (Exact periodic solutions in Methods). In B, the patterning region is the one below the green dashed line. B and D show that patterning becomes forbidden as 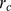 increases when cis-activation is acting. C and D show that patterning is enabled above a minimal cis-interactions strength 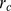 when there is cis-inhibition. Other parameter values: 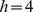 for all panels, 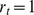 and 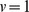 for A, 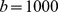 for A–B and D, 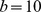 for C.

From theoretical arguments it has been shown that the intercellular positive feedback mediated by trans-interactions is a sufficient mechanism for spontaneous pattern formation [13]. This feedback amplifies small differences in ligand and signal levels between cells and drives a mostly periodic lateral inhibition pattern composed of two cell types (Fig. 3A)[13]. This type of periodic pattern arises spontaneously for a large range of trans-interactions strengths (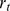) and above a minimal ligand inhibition strength (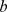) (Fig. S2 and Methods) [13]. The pattern solution exists and is stable in an even larger region of the parameter space, with a minimal yet lower ligand inhibition strength (Fig. S2 and Methods) [10], [56].

From the effective circuit architectures (Fig. 2F), we may propose that cis-activation can inhibit pattern formation. Cis-activation drives a negative feedback loop within cells which could damper the amplification of differences between precursor cells driven by trans-interactions. In contrast, we may expect that cis-inhibition can promote patterning as it can enhance amplification of precursor differences by driving an additional positive feedback loop within cells (Fig. 2F). This latter expectation is in agreement with previous computational studies on cis-interactions that do not trigger a signal [25], [29], [33], [35].

We first evaluated the case of efficient cis-signaling (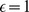) driving cis-activation whatever the strength and amount of trans-interactions. We explored extensively the parameter space to characterize where lateral inhibition patterning occurs (Methods). We chose a regime for which the lateral inhibition pattern can arise for a wide range of trans-interactions strengths (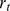) in the absence of cis-interactions (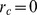; Fig. 3B). When cis-interactions are added (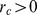), pattern formation becomes forbidden (Fig. 3B). At very weak cis-interactions strengths, pattern formation is still possible albeit in a reduced range of trans-interactions strengths. We conclude that cis-activation inhibits patterning.

Following the same procedures, we evaluated whether pattern formation is promoted by cis-inhibition. To this end, we analyzed the well-known case of cis-interactions which do not elicit signaling (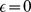), driving always cis-inhibition. In this case, it is known that cis-inhibition facilitates patterning by allowing it for graded trans-signaling and graded ligand inhibition (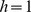) [25] (Fig. S3A). We found that cis-inhibition facilitates spontaneous patterning as well for low ligand inhibition cooperativities (Fig. S3B). At higher cooperativities, the analysis showed that cis-inhibition promotes patterning too by reducing the minimal ligand inhibition strength (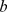) required for patterning (Fig. 3C). Cis-inhibition can have a detrimental effect as well (Fig. 3C). When the strength of cis-interactions is too high compared to trans-interactions strength (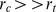), the coupling between cells becomes less relevant, impeding patterning. Yet, if the trans-interactions strength increases, patterning is enabled (Figs. 3C and S3A–C).

We finally evaluated a more complex scenario in which cis-signaling switches from cis-activation to cis-inhibition when trans-interactions strength (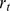) increases (Fig. 3D). Our results show that in the cis-activation regime, pattern formation is inhibited, since an increase in cis-interactions impedes pattern formation. In contrast, in the cis-inhibition regime, pattern formation is facilitated since a minimal value of cis-interactions strength (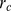) enables spontaneous patterning. These results confirm the existence of distinct regulatory roles of cis-signaling (cis-activation *versus* cis-inhibition) as a function of the amount of trans-interactions as well as the different effect each of them has on lateral inhibition patterning. Taken together, the results suggest that the effect of cis-signaling on patterning can be simplified to that of the regime in which it is acting.

### Cis-inhibition can modulate patterning and enhance multistability

**Figure 4:**
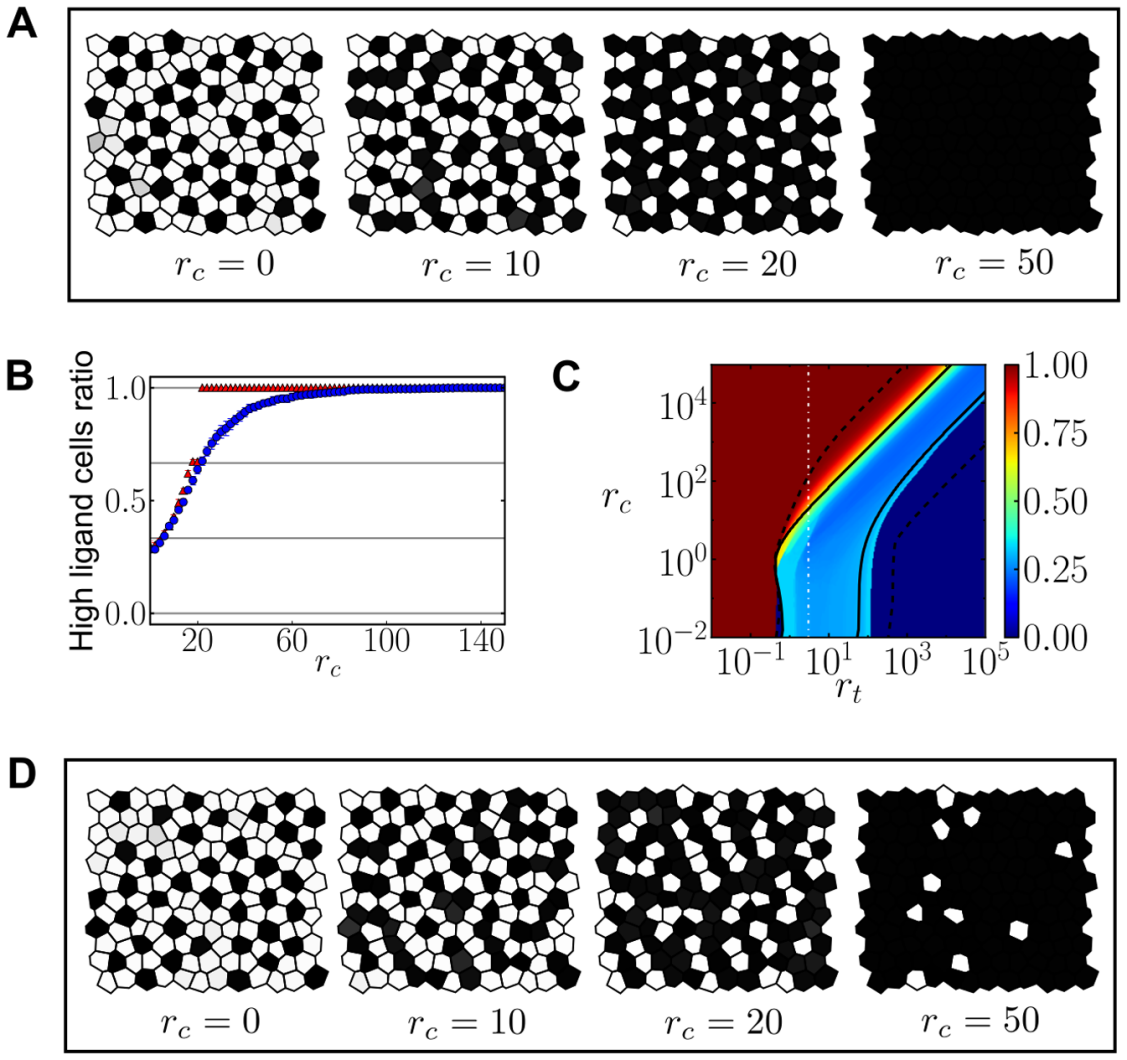
Cis-inhibiting interactions increase the ratio of high-ligand expressing cells. (A) Stationary patterns of ligand levels arising from precursor cells with small initial variability between them for different inhibiting cis-interactions strengths 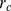. Color code as in Fig. 3A. (B) Ratio of stationary high-ligand fated cells as a function of the cis-interactions strength 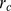 when precursor cells show small (red triangles) and large (blue circles) initial variability between them. (C) Density plot representing the ratio of high-ligand cells in a tissue arising from precursor cells exhibiting large initial variability. Solid and dashed lines as defined in Fig. 3B–D. White vertical line is drawn for indicating the value 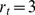 along which simulations are performed in panels A, B and D. (D) Stationary patterns of ligand levels arising from precursor cells with large initial variability between them for different inhibiting cis-interactions strengths 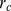. In B–C panels, cells are considered high-ligand fated cells when its ligand level is over the threshold of 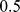. Parameter values: 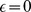, 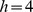, 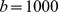 and 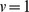 for all panels. Similar results are found for 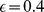 (Fig. S5). In B, each point comes from the average of 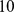 numerical integrations of the dynamics on a lattice of 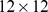 irregular cells starting at different initial conditions. In C, the results correspond to numerical integration of the dynamics performed over a lattice of 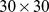 perfect hexagonal cells.

We next evaluated which patterning features arise in the cis-inhibition regime. Cis-inhibition can make cells worse receivers of inhibition [14], [20], [26], [57]. This is confirmed in our model by evaluating the change in the threshold level of ligand activity required to drive ligand inhibition in an adjacent cell when cis-interactions are added in the receiving cell (Methods, Fig. S4). When cells become worse receivers of inhibition we can expect the ratio of high-ligand expressing cells to increase. Simulations results confirm cis-inhibition can increase the ratio of selected precursor cells (Fig. 4A for 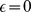 and Fig. S5 for 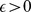). The strength of cis-interactions (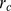) and the fraction between cis and trans-interactions strengths (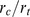) become a control parameter for this ratio (Figs. 4B,C). When precursor cells exhibit large random initial variability between them in ligand and signal levels, the strength of cis-interactions can increase more gradually the ratio of high-ligand expressing cells (Figs. 4B–D).

**Figure 5:**
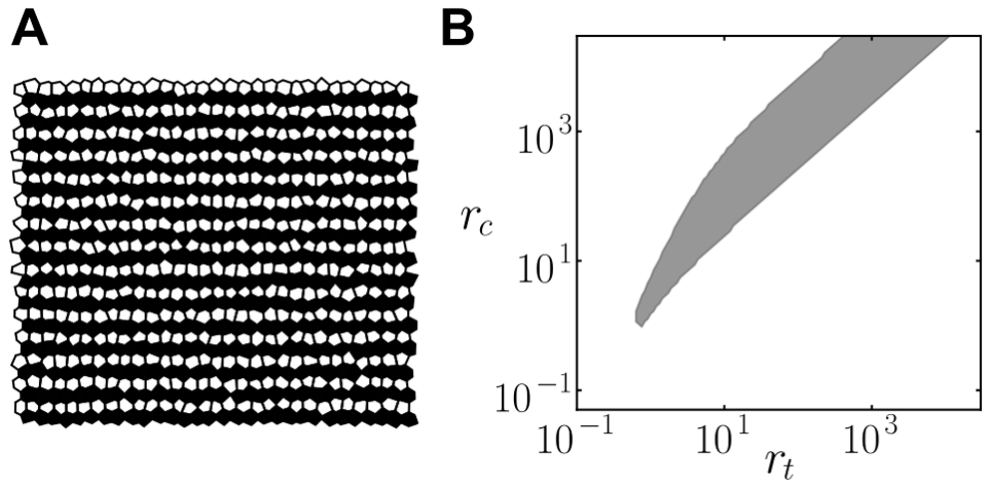
Cis-inhibiting interactions facilitate other periodic patterns to form. (A) Stationary stable stripped pattern of ligand levels that is a stable solution of the dynamics to small perturbations. Color code as in Fig. 3A. (B) Region (gray) where the pattern of stripes on a regular hexagonal array is a stable solution of the dynamics to small perturbations (Methods and Text S1) in the parameter space of cis and trans-interactions strengths 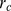 and 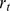. Parameter values: 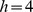, 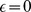 and 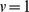 for all panels and 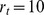 and 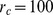 for panel A. The stripped pattern appears also for 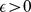 in the cis-inhibition regime (data not shown).

These results showed that cis-inhibition can enable a new regular salt-and-pepper pattern with 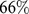 of cells highly expressing the ligand (Figs. 4A, S5). This pattern has the periodicity of the lateral inhibition pattern. However, the ratio of selected high-ligand expressing cells is complementary to it. Since our results show that cis-inhibition can modulate the threshold for lateral inhibition and thereby the ratio of selected precursors, we wondered whether it can enrich patterning and drive additional periodic patterns. By using a combined analytical-computational approach (Methods), we searched across the parameter space of cis-interactions (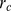) and trans-interactions strengths (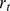) whether and where different periodic patterns composed of two cell types were stable solutions of the dynamics. We chose to search for three different types of patterns that involve different numbers of selected precursors and spatial organizations (Fig. S6). One of them is the salt-and-pepper pattern of Fig. 4A with a 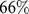 ratio of selected precursors (Fig. S6A). Another one is a salt-and-pepper pattern too but with a different periodicity and a 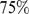 ratio of selected precursors (Fig. S6B). The third chosen pattern is stripped with 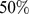 of cells expressing high-ligand levels (Fig. S6B). Our study showed that high enough cis-interactions strengths (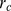) enable the emergence of these patterns with high numbers of precursor cells (Figs. 5, S7, S8 and S9). In the absence of cis-interactions and for low cis-interaction strengths (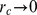), the salt-and-pepper spatial organizations can persist but with much lower numbers of selected precursors (33% and 25%, Figs. S7, S8). In contrast, cis-inhibition with high enough cis-interactions strengths (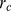) enable the spatial organization of precursor cells within stripes (Figs. 5, S9).

Additionally, we found that for high cis-interactions strengths (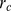) all these patterns are stable, *i.e*. there is multistability of pattern states (Figs. S7–S9). Hence, precursor cells could potentially become organized in any of them and should choose which pattern to form (Palau-Ortin *et al*., unpublished).

### Cis-inhibition allows localized patterning highly sensitive to the precursor state

**Figure 6:**
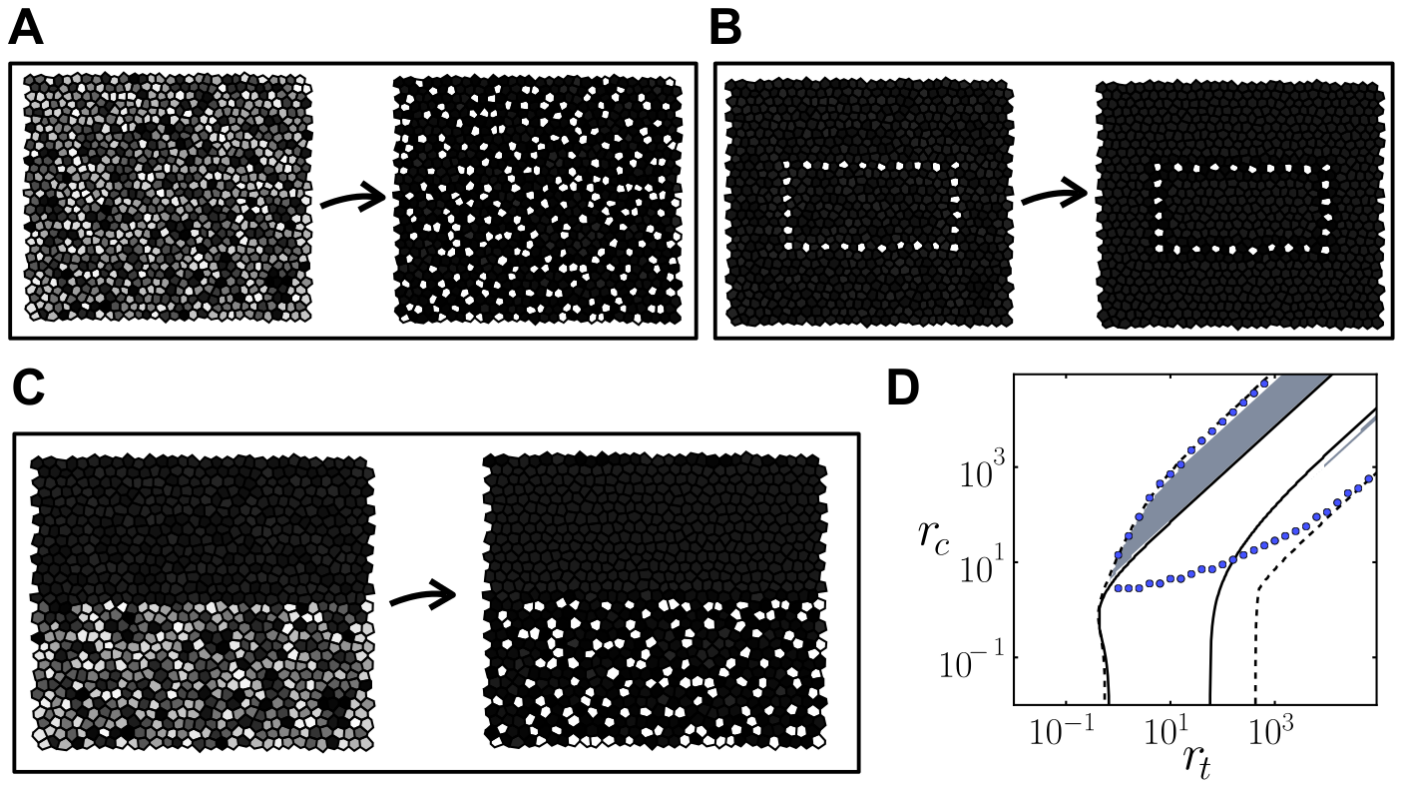
Cis-inhibition allows pattern localization. (A–C) Initial (left) and stationary (right) patterns of ligand levels at high cis-interactions strengths 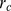 in the cis-inhibition regime for different initial conditions: (A) all precursor cells have large initial random variability, (B) few precursor cells, distributed along a rectangle, have initial low ligand levels and (C) precursors within the top half of the tissue have initial high ligand levels and small variability, while precursors at the bottom half show large initial random variability in the level of ligand. In A–C, the final pattern strongly depends on the pattern formed by precursor cells. In (C) the pattern arises in a localized region (bottom half) and does not expand. (D) Region where localized patterns are found in a regular hexagonal array (gray) in the parameter space of cis and trans-interactions strengths 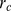 and 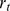. Blue circles enclose the region for cell-autonomous bistability, where two states are linearly stable, according to simulation results (Methods). Solid and dashed lines as in Fig. 3B–D respectively. Parameter values: 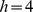, 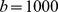, 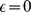 and 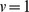 for all panels and 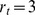 and 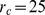 for (A–C).

As shown in Fig. 4D, stable patterns without an obvious periodicity can arise too for high strengths of cis-inhibiting interactions (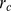). This occurs when precursor cells show large initial random variability between them, in agreement with [25]. We evaluated whether a high sensitivity to the initial state of precursor cells was causing the absence of periodicity. The results show that the finally formed stable pattern has strong similarity to the initial state of precursor cells (Fig. 6A–B). Simulations across the parameter space using precursor cells with large initial random variability between them confirmed that the pattern being formed is quite random as the state of precursors cells is for high cis-interactions strengths (Fig. S10). In contrast, large initial random variability between precursor cells dynamically evolves to more regular and periodic patterns of two cell fates for lower cis-interactions strengths (Fig. S10).

Simulations results indicated that the patterns exhibiting high sensitivity to the initial random state of precursor cells keep spatially localized without spreading to the rest of the tissue (Fig. 6C). This absence of spreading is in sharp contrast with the dynamics of nucleating patterns driven only by trans-interactions. Nucleating patterns invade the rest of the tissue that is under lateral inhibition Notch dynamics through a traveling wave [58], [59]. The localized patterns we find remain where they arise and do not spread despite the adjacent tissue is under lateral inhibition dynamics too. We searched across the parameter space of cis-interactions (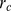) and trans-interactions strengths (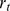) where pattern localization occurs (Fig. 6D). This pattern localization occurs in a specific region of the parameter space where cis-inhibiting interactions are dominant (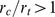). In addition, in this region there are many different stable pattern solutions and a homogeneous linearly stable state that impedes spontaneous patterning from small initial variability between precursor cells (Figs. 6D, S7–S9). These results suggest that strong cis-interactions performing cis-inhibition enrich patterning from precursor cells that show large initial variability between them. The arising patterns keep localized within the tissue and are reminiscent of the initial states of precursor cells.

### Cis-inhibition can drive cell-autonomous bistability

We reasoned that cis-driven dynamics at the cell-autonomous level could be relevant for the phenomenon of localized patterning. Specifically, we wondered whether cis-inhibition could drive bistability of distinct ligand and signal level states in isolated cells. To evaluate it, we considered the role of cis-inhibition in the dynamics of single isolated cells that have a primary source of Notch signal (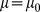, being 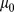 a constant, see Text S1). This primary signaling source could be ligand-independent.

**Figure 7:**
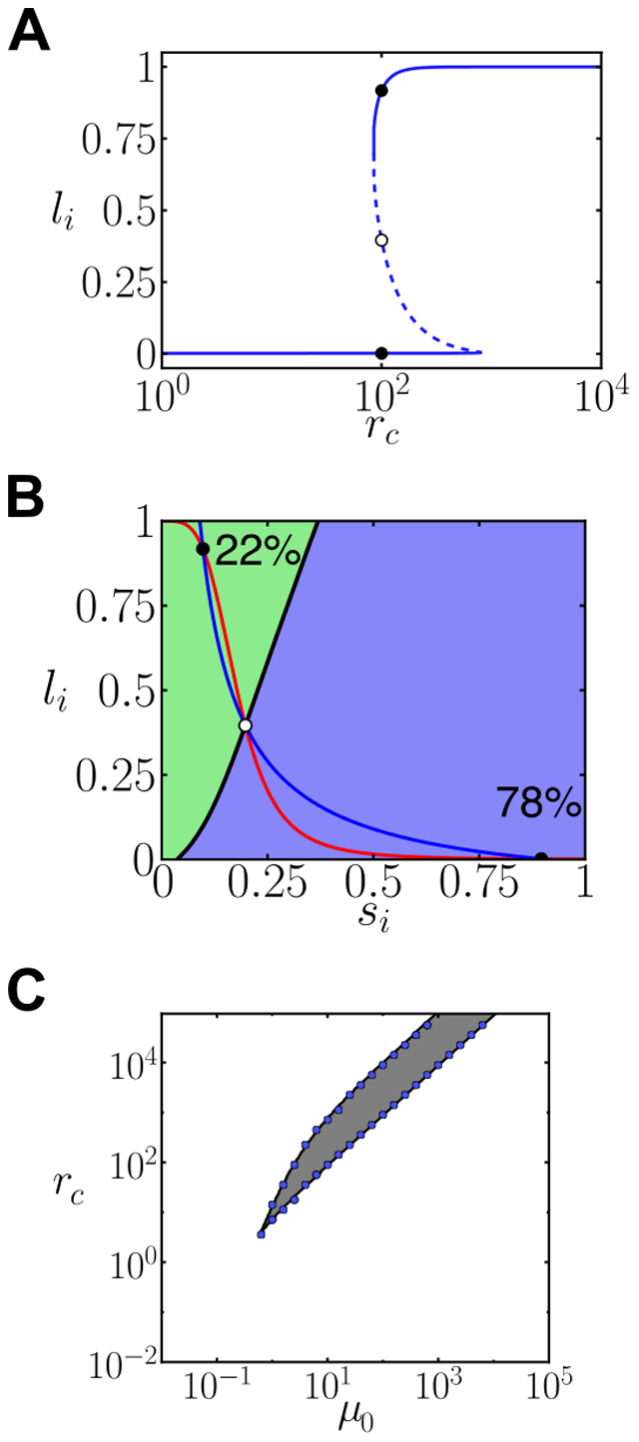
Cis-inhibition with a primary Notch signaling source creates cell-autonomous bistability. (A) Stationary ligand level as a function of the cis-interactions strength 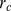 for 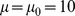 and 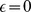. Solid lines denote linearly stable solutions, dashed lines indicate linearly unstable solutions. Black dots refer to the stationary ligand levels for 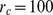. (B) Nullclines diagram showing the three possible solutions at 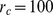. The blue and red lines represent the nullclines. The continuous black line is a separatrix, which divides the parameter space into two basins of attraction of the two stable solutions. Percentages indicate the fraction of cells reaching the corresponding stable state computed from 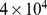 cells with initial random uniform levels of ligand. (C) Phase diagram showing the cell-autonomous bistability region zone where two states are linearly stable. The gray area is the theoretically computed region, and the blue circles correspond to simulation results (Methods). Parameter values: 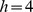, 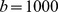, 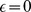 and 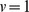 for all panels. These results can also be obtained for 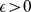 in the cis-inhibition regime (data not shown).

Our results show that cis-inhibition can drive cell-autonomous bistability when a primary signaling source is present (Fig. 7A,B, Methods). This bistability drives similar cell types to the ones found in lateral inhibition patterning: cells are expected to be either on a high-ligand expression state or in a low-ligand expression state with opposite signaling state (Fig. 7A,B). Bistability of cell fates requires a minimal amount of cis-interactions (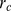) and of primary signaling source (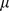; Fig. 7C).

We evaluated the existence of this cell-autonomous bistability when the amount of primary signal corresponds to the signal that trans-interactions drive for the homogeneous state of equivalent cells (Methods). For this primary signal, bistability arises for high cis-interactions strengths (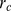) and encloses the region where patterns keep localized (Fig. 6D). This result suggests that cell-autonomous bistable dynamics arising from cis-inhibition may be relevant for the phenomenon of non-periodic pattern localization.

## Discussion

### Competition for signaling: a switch from cis-activation to cis-inhibition

Several experimental evidences support the existence of signaling driven by cis-interactions [39]–[43]. In this work we have theoretically characterized the effect of cis-signaling in different contexts. We found that a switch from cis-activation to cis-inhibition (or *vice versa*) arises. The switch can occur by quantitatively changing the signaling sources; either by changing the amount of the primary signaling source (*e.g*. trans-interactions), or by modulating the ratio between the signaling efficiencies of each source. As a result, phenotypes involving a reduction of Notch signaling when the ligand is increased within a cell (cis-inhibition) can be compatible with cis-signaling.

The results show that cis-signaling can drive opposed capabilities to the patterning process, each arising on the different regulatory regimes of cis-activation and cis-inhibition. Cis-signaling acting as cis-activation creates a negative intracellular feedback loop that inhibits pattern formation. On the other side, cis-signaling acting as cis-inhibition creates a positive intracellular feedback loop facilitating patterning. This regime promotes patterning as cis-inhibition driven by null cis-signaling does [25], [33], [35].

The switch from cis-activation to cis-inhibition exemplifies a case of competition leading to a complex dynamical output: two signaling sources – the primary and the cis sources – with different efficiencies and competing for the same substrate – the Notch receptor – can result in a cis-productive signaling that drives cis-inhibition. Such effect is reminiscent of the behavior of full and partial pharmacological agonists, where a partial agonist can act as a competitive inhibitor of a full agonist [60]. It is also an example of complex regulation in which the interplay between different components changes the regulation performed by one of them [61].

This notion can be extrapolated to any competition between signaling sources that share the same receptor. When different ligands (canonical or not) bind the same type of Notch receptor but drive signaling with different efficiencies, one can expect competition between them and switches of regulatory roles [62] (Jelena *et al*., unpublished). This approach could also help to decipher the controversial roles of different non-canonical factors that bind to Notch and present both activatory and inhibitory effects on Notch signaling. For instance, this is the case of the Dlk1/2 non-canonical ligands [3], [63] and the proteins MAGP1/2 [45], [64]. Different competition events have already been shown to drive significant regulatory effects in other signaling pathways (see for instance [65]–[70]).

In addition, our approach provides a definition for the ratio of signaling efficiencies coming from different sources that share the same receptor. This ratio is defined by the signaling rates and by the stability of the signaling sources. This definition is relevant to determine the regulatory role each signaling source drives on the overall signaling.

#### Cis-inhibition as a modulator of the ratio of selected precursor cells

From experimental grounds it has been already pointed out that cis-inhibition can drive cells to become worse receivers [14], [20], [26], [57]. This effect can be obtained from our model too and enables cis-interactions to increase the number of high-ligand expressing cells. Cis-inhibition is expected also to drive cells to become worse signal senders by sequestering the ligand. Albeit this aspect is not considered in our simplified model, this effect should increase the ratio of cells reaching the high-ligand fate too. We have checked that a more complex model involving ligand sequestration similar to Sprinzak *et al*. (2010) (Methods and Text S1) also shows an increase in the ratio of high-ligand expressing cells. Consistent with our simplified model, competition between signaling sources in the complex model also yields switches of the regulatory role of cis-interactions (Fig. S11, Methods). The results confirm the increase in the ratio of high-ligand fated cells with the strength of cis-interactions in the cis-inhibition regime (Fig. S12). This complex model indicates that this increase occurs too when there is no cooperativity in the inhibition of the ligand (*i.e*. for 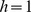 in Eq. S4a of Text S1, Fig. S12).

It has been reported that Lunatic Fringe knockdown produces an increase in the neurogenesis ratio in the hindbrain of zebrafish embryos [71]. This could be an example of the cell-type ratio modulation we find in our simulations, since Fringe potentiates Delta-Notch trans-interactions [72] and could inhibit the cell-autonomous association of Delta and Notch [20].

The strength of cis-inhibiting interactions is not the only potential modulator of the ratio of high-ligand expressing cells. Specifically, different theoretical approaches that do not take cis-interactions into account have reported other components that can modulate this ratio through changes in the level of the threshold that drives inhibition of the ligand [73], [74]. Also, it has been shown that higher rates of Delta production can drive a graded increase of high-Delta cells, which was validated experimentally [75].

Noteworthy, Notch signaling dynamics *in vivo* can select a different ratio of high-ligand cells in different contexts [76]–[81]. Our results suggest that cis-inhibition could underlie the selection processes that involve high ratios of selected cells.

#### Cis-inhibition potentiates multistability and enriches patterning

Multistability can enable the change of fate of cells and it has been widely evaluated in the context of single cells, specially in bacteria and stem cells. A recurrent circuit topology that shows multistability and participates in stem cell renewal and differentiation is the toggle switch with auto-activation [82]–[84]. Auto-activation in this dynamics facilitates multistability [82]. Herein we show that lateral inhibition Notch dynamics with cis-inhibition can be described with an effective topology that corresponds to a toggle switch with auto-activation (Fig. 2F). In this effective topology, cis-inhibition drives auto-activation and facilitates multistability, in agreement with the effect of auto-activation in cell-autonomous toggle switches. This is reminiscent to the reported multistability in the ommatidia formation in *Drosophila* eye, which has been proposed to be driven by an auto-activatory feedback loop due to Atonal [85].

At the cell-autonomous level, we find that cis-signaling can drive bistability of ligand and signaling states when a basal cell-autonomous activity of Notch is present and cis-inhibition is acting. In this case, the effective circuit topology corresponds to a positive feedback loop that involves a mixed-feedback loop [86], [87]. The bistability regime is confirmed and becomes more prominent in the more complex model (Fig. S13, Methods). The prediction of cell-autonomous bistability due to cis-inhibition could shed light to new functions of Notch in single cells. Recently, cell-autonomous bistability in Notch has been identified in the context of colon cancer stem cells [88]. In particular, it has been shown that the sequestering of mRNA Notch1 by the tumor suppressor microRNA miR-34a drives cells with Notch signal bimodality [88].

Simulation results show that cis-inhibition enables the spatial localization of patterns, which do not propagate spontaneously on the entire tissue. This could correspond to a wave-pinning phenomenon [58]. Typically, wave-pinning arises in discrete dynamical systems when the coupling between the discrete units is below a critical strength [89]. In our scenario, the critical coupling would be related to cis *versus* trans-interactions strengths (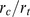 ratios). Moreover, the most disordered patterns, with high-ligand cells adjacent to each other [25], appear in the region where localized patterning occurs. In such regions of the parameter space, the final pattern strongly depends on the initial precursor state. This dependence on the precursor state is reminiscent of the directionality provided by cis-inhibition in the differentiation of R1/R6/R7 precursor photoreceptor cells in the *Drosophila* eye [26]. Together, these results suggest that cis-inhibition can enrich patterning by enabling additional modulations of cell fate decisions.

## Methods

### Model formulation within an irregular cellular array layout

The simple model phenomenologically includes the competition between cis and the primary signaling sources for the Notch receptor and the inhibition of Notch signaling on the ligand. It is based on the approach introduced by Collier *et al*. [13] for lateral inhibition dynamics through trans-interactions. We included competition such that it is in agreement with a more biochemical reaction-based approach (see Complex model below). The simplicity of the simple model strongly facilitates the vast exploration of different patterning regions in the parameter space.

The weighted average of non-dimensional ligand concentration, 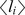, appearing in Eq. 1 through 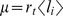 due to trans-interactions, describes the interactions between adjacent cells on a two-dimensional irregular array of cells:
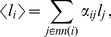
(6)with 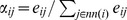, being 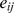 the length of the cell membrane edge shared by adjacent cells 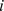 and 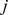, and the summation involves all cells adjacent to cell 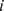 (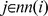) [90]. We constructed an irregular two-dimensional array of cells with periodic boundary conditions as in [10] with irregularity parameter 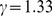 (see Fig. S3 in [10]). First, we generated an irregular distribution of points on a plane starting from a perfect triangular lattice and considered periodic boundary conditions by surrounding the array of points with equivalent arrays. Second, a Voronoi tessellation was created around these points using Mathematica's Computational Geometry Package (Wolfram Research, Inc. (2008), Mathematica, Version 7.0, Champaign, IL, USA).

### Complex model

The Complex model takes into account the dynamics of the Notch receptor and of the complexes formed by receptors and ligands (see Text S1 for all reactions, model equations and further details). It includes receptor and ligand inactivation through proteolytic cleavage [25], [35] and it does not make assumptions regarding the capability of sending and receiving signals (*e.g*. when cis-interactions are acting, signal sending cells are not necessarily refractory to receive inhibitory signals from its neighbors). In this Complex model, the dynamics of the free receptor (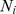), trans and cis-formed receptor-ligand complexes (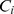 and 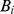 respectively), and Notch signal 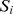 in cell 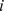 when the primary signaling source is due to trans-interactions read:
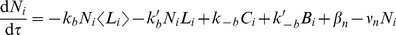
(7)




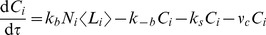
(8)




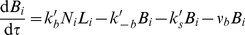
(9)




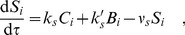
(10)


where the variables are in dimensional units and 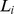 is the free ligand in cell 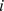, whose dynamics are detailed in Text S1. 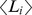 is given by Eq. 6 applied on species 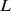. 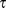 is the dimensional time. The binding and unbinding dynamics of Notch receptors with its ligand in trans and in cis have rates 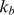, 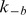 and 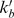, 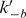, respectively. Notch production (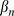) and degradation (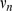) are also taken into account. Trans and cis complexes, 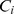 and 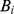, have degradation rates 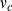 and 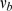, respectively. The model does not detail the overall mechanism by which trans-interactions drive signal activity. Instead, it assigns a rate 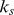 to the proteolytic cleavage of the trans complex and the ultimate release of Notch signal 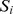. The model also considers the case in which cis-interactions drive Notch signaling. We implemented it by taking into account the argued mechanism for cis-signaling [40], so that the release of Notch intracellular domain would also occur for cis complexes (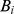). We set this step to occur at rate 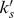. By taking 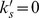, the above equations account for the usual scenario of cis-interactions that sequester the receptor and drive no signaling.

Notice that the stationary solution of Eqs. 1–3 is the same function of 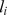 and 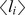 as the stationary solution of Eqs. 7–10, which reads (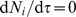, 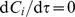, 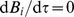, 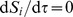):
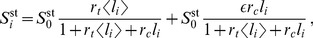
(11)with 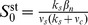, 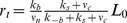, 
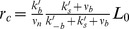
 and 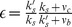. 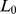 is a characteristic dimensional concentration of ligand (*i.e*. 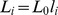). The first term on the right-hand side corresponds to the stationary primary signaling driven by trans-interactions whereas the second term is the stationary signaling driven by cis-interactions.

We define the efficiency of each source as the ratio of success to signal of the receptor-ligand complexes. This efficiency corresponds to 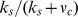 for the primary signaling source and to 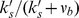 for cis-interactions. Therefore, 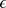 parameter (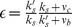) is the relative efficiency of the cis-driven source compared to that of the primary signaling source. From Eq. 11 it can be seen that 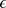 corresponds as well to the ratio of maximal saturated stationary Notch activity driven by cis-interactions over that one driven by the primary signaling.

The equations of the Complex model for single isolated cells with ligand-independent and cell-autonomous primary signaling sources are detailed in Text S1.

### Evaluation of the regulatory role of cis-interactions

We defined the regulatory role of cis-interactions (cis-inhibition or cis-activation) through the (negative or positive, respectively) change in Notch signal dynamics within a cell when its ligand content increases, 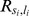:
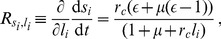
(12)where the result of the derivative for the model described by Eqs. 1–3 is indicated. Cis-inhibition is defined as a decrease in Notch signaling when the ligand content increases within the same cell, *i.e*. 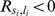, whereas cis-activation corresponds to an increase in Notch signaling, *i.e*. 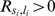. Based on the above expression for 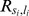, cis-interactions drive cis-inhibition when 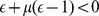. Therefore, the condition for cis-inhibition to happen can be re-written as inequality 5:
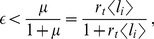
(13)where 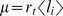 for trans-interactions has been introduced in the last right-hand side term. This inequality states that cis-inhibition occurs when the maximal (saturated) signaling driven by cis-interactions is lower than the signaling driven by the primary source when acting alone (Fig. S1). Notice that this criterion for the cis-regulatory role is independent of 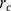 (Fig. S1C).

Eq. 13 gives the regulatory role of cis-interactions in the Complex model at the steady state as well (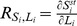) with 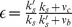 and 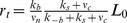. Accordingly, for no cis-signaling (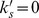) cis-interactions perform cis-inhibition, whatever the context and additional parameter values. For 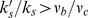 (*i.e*. 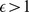), cis-interactions perform always cis-activation. For 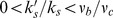 (*i.e*. 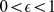) a switch from cis-activation to cis-inhibition can occur as the amount of trans-interactions increase. Noteworthy, when the receptor-ligand complex formed by cis-interactions is more unstable than the complex formed by trans-interactions (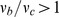), it can drive cis-inhibition even if it signals faster than the trans complex (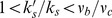).

From the model equations, it can be readily seen that the switch of regulatory role can only take place in the non-linear regime of the signaling function (Eqs. 1, 2 and 11). Notice that this regime does not require saturation of the Notch receptors. When the primary source is acting in the linear regime (*i.e*. 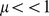), the addition of ligand within the cell does not reduce the primary signaling since there is no competition for the Notch receptor. As a result, cis-signaling always drives cis-activation in this linear regime.

The inequality arising for isolated cells with cis-signaling and a primary signaling source is detailed in Text S1 (Fig. S11B).

### Linear stability analysis (LSA)

Linear stability analysis [13], [56] has been applied to Eqs. 1–4 to determine in which regions of the parameter space spontaneous patterning occurs. We defined spontaneous patterning as the process that drives pattern formation from a linear instability of the homogeneous initial state through small non-homogeneous perturbations (*i.e*. when small initial variability between precursor cells becomes amplified) [91]. LSA enabled us to make analytic predictions of how the pattern formation capabilities of the system would be changed by cis-interactions. LSA indicated that cis-interactions do not change the fastest growing mode, and hence the periodicity of the pattern is expected to be the same as in the absence of cis-interactions for spontaneous patterning (Text S1). LSA indicated that spontaneous patterning in a regular hexagonal array of cells with periodic boundary conditions would happen when (see Text S1 for details and Fig. S14)
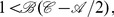
(14)being
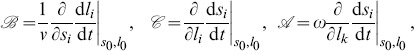
(15)where 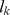 is the ligand level in a neighboring cell to cell 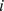, 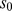 and 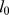 are the homogeneous steady states for the multicellular system (*i.e*. the solutions of 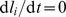, 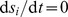 for 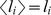) and 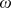 is the number of nearest neighbors in a hexagonal cellular array (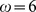). 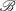 measures the strength of ligand repression at the homogeneous stationary state and verifies 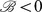. 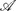 measures the strength of trans-activation and verifies 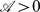. Notice that 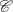 is 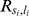 computed at the homogeneous steady state. Therefore, when cis-inhibition (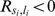) is acting at such homogeneous state then we have 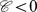. 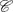 measures the strength of cis-inhibition (when 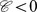) and of cis-activation (when 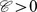).

According to inequality 14, cis-inhibition (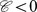) facilitates patterning (Fig. S15). In contrast, cis-activation (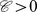) inhibits patterning (Fig. S15). Inequality 14 was also used to evaluate where spontaneous patterning can emerge in the 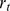
–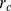 parameter space. These results are depicted by solid lines in Figs. 3, 4 and 6. The use of the simple model of Eqs. 1–4 enabled a vast exploration across the parameter space. We checked several of the regions obtained by LSA with numerical simulations (Text S1, and Fig. S16 as example).

### Exact periodic solutions

We evaluated the 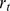
–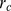 parameter space regions where the lateral inhibition pattern is a stable solution of the dynamics defined by Eqs. 1–4. We also evaluated whether other periodic patterns are stable solutions of these dynamics. This analysis was strongly facilitated by the use of the simple model. To this end, we extended a method we previously introduced [56] to our system and to new periodic patterns. We considered periodic patterns composed of only two different cell types: cell type 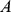 and cell type 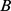. According to dynamics given by Eqs. 1–4, the stationary state values (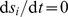, 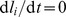) of these two cell types are:
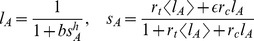
(16)




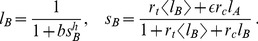
(17)


Based on the periodicity of the pattern, we imposed which is the neighborhood of cell types each cell type interacts with:
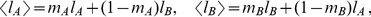
(18)where 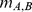 is the ratio of 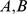-like cells neighboring to the 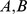-cell type. For the common lateral inhibition pattern we have 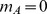 and 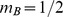. The 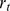
–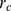 parameter boundary regions enclosing the region where this lateral inhibition pattern is a stable solution of the dynamics are depicted with dashed lines in Figs. 3, 4 and 6. The 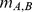 values for other periodic patterns are as follows. For an additional salt-and-pepper pattern (Fig. S6B) we have 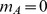 and 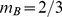. For the stripped pattern 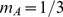 and 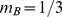 (Fig. S6B).

Solutions of Eqs. 16–18 were found using NSolve from Mathematica and also through custom made programs using the bisection method. Stability of solutions was evaluated computationally by numerical integration of the dynamics with patterned initial conditions (see below and Text S1). Together, these results show that the parameter space region where the pattern with the periodicity of the lateral inhibition pattern is stable is the largest one and contains the regions where the other patterns are stable (Figs. S7–S9).

### Threshold for lateral inhibition

We evaluated how cis-interactions within a cell (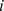) change its capacity to receive the inhibition from adjacent cells. We termed 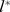 the threshold for lateral inhibition and defined it as the ligand activity in adjacent cells that drives the inhibition of the ligand in cell 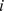. This inhibition of the ligand was defined as having a production rate of ligand activity 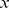 times (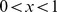) the maximum production rate, which is 1 for Eq. 4. Therefore, according to Eq. 4, a production rate 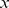 of ligand occurs for 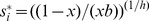. We then computed which ligand activity in adjacent cells 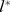 is required to drive a stationary signaling 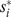 within cell 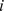 if this cell has a ligand activity 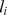 which drives cis-interactions (*i.e*. we isolated 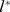 from 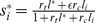 according to Eqs. 1–3). Taken together we obtain that the threshold for lateral inhibition is:
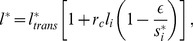
(19)where 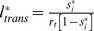 is the threshold for lateral inhibition in the absence of cis-interactions. The above equation indicates that cis-interactions increase this threshold (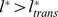) making cells worse receivers of inhibition when cis-inhibition is taking place (Fig. 4A–C).

### Cis-driven cell-autonomous bistability

We evaluated whether the positive feedback generated by cis-inhibition with a competing primary signaling source in isolated cells (Fig. 2E) is sufficient to drive cell-autonomous bistability. To this end, we computed the steady state solutions (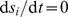, 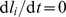) for Eqs. 1–4 when 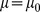. Linear stability of these solutions was given by
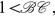
(20)where 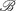 and 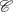 are defined as in the LSA section in Methods but are evaluated at the steady state solutions. Analysis of the nullclines (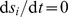, 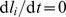) indicates that cis-inhibition is required to have bistability (both nullclines need to be decreasing functions). We extensively explored in the 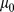
–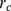 parameter space where bistability occurred with LSA, and corroborated it with numerical simulations (Fig. 7C). In Fig. 6D we used 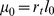 being 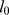 the homogeneous steady state for the multicellular system. All these simulations were performed for 400 cells with random initial conditions. The region of bistability in the phase space was delimited similarly than the LSA regions (Text S1). The stability of the bifurcation branches in Fig. 7A was checked with simulations of 900 cells with small fluctuations around the different branches.

Cell-autonomous bistability in the Complex model was analyzed through nullclines analysis and numerical integration of the dynamics. In this case, bistability arises too in the absence of cooperativity in ligand inhibition (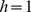).

Fixed points were computed with Mathematica with the NSolve function and with custom made programs with a bisection method.

### Numerical integration of the dynamics

We integrated Eqs. 1–4 with custom made programs using a Runge-Kutta fourth-order algorithm [92] with a time step of 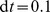. The Complex model for the multicellular and single cell system was integrated with Mathematica by using the NDSolve function.

To evaluate which patterns were formed and their stability we used three types of initial conditions. (1) Precursor cells (*i.e*. cells at their initial condition) show small variability between them: for each molecular species 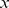, 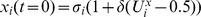 being 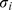 the homogeneous steady state for the specie 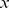, 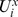 a uniform random number between 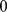 and 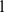 and 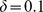. (2) Precursor cells show large random variability between them: 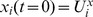, unless otherwise stated. (3) Precursor cells already form a regular pattern, with small variability: 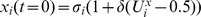 being 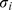 the steady state pattern solution and 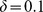. We performed numerical integration of the dynamics for different parameter values defined on a logarithmic mesh across the 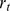
–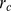 parameter space. Simulations were stopped when the steady state was reached.

## Supporting Information

Figure S1
**The regulatory role of cis-interactions when acting together with trans-interactions.** (A–C) Signal production rate in cell 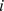 due to (left) trans 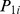 and (middle) cis-interactions 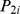, and (right) total signal production rate 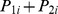 as a function of the ligand level 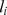 within the cell. (Left) 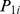 decreases with 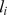 for all parameter values because cis-interactions drive competition for the Notch receptor. 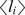 value is set at the homogeneous fixed point. (Right) 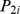 always increases with 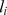 since we impose that cis-signaling on its own activates Notch signaling. (Right) 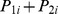 can be either a decreasing or an increasing function of 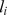. This indicates the regulatory role of cis-interactions. Cis-inhibition occurs when 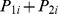 decreases with 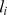, whereas cis-activation is acting when 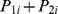 increases with 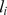. (A) Signal production rates for different ratios between cis/trans signaling efficiencies 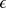: 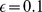 (gray solid line), 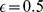 (black solid line) and 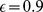 (dashed line). The regulatory role changes from cis-inhibition to cis-activation as 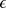 increases. (B) Signal production rates for different trans-interactions strengths 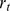: 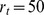 (gray solid line), 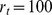 (black solid line) and 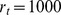 (dashed line). The regulatory role changes from cis-activation to cis-inhibition as 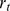 increases. (C) Signal production rates for different cis-interactions strengths 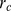: 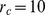 (dashed line), 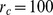 (black solid line) and 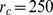 (gray solid line). The regulatory role does not change with 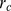. This is because neither 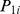 at 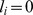 (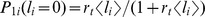) nor the saturated value of 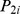, 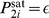, depend on 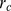. Parameter values are 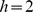, 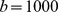, 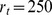, 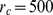 if not indicated otherwise, (B) 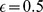 and (C) 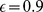.(TIFF)

Figure S2
**Results in the absence of cis-interactions (** 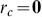 **).** (A) Scheme of interactions as in [13] of two cells that inhibit each other through Notch-mediated lateral inhibition. Black (blunt red) arrows denote activation (inhibition). Notice the positive intercellular feedback loop. (B-C) Phase diagrams in the parameter space of ligand inhibition strength 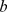 and trans-interactions strength 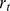 for (B) high (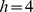) and (C) low (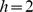) cooperativity in ligand inhibition. The blue region in (B) is where the homogeneous state is linearly unstable. This is the region of spontaneous patterning, where the lateral inhibition pattern can arise from the amplification of small differences between precursor cells, as described in [13]. The region above the dashed line is where the pattern solution (with the periodicity shown in Fig. 3A) is an exact stable solution of the dynamics [56]. Above the dashed line and below the blue line in panel B both the homogeneous state and the lateral inhibition pattern are stable solutions of the dynamics (*i.e*. it is a bistable region). The continuous and dashed lines in (B) have been shown in [10]. Spontaneous patterning does not occur at low cooperativities (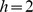).(TIFF)

Figure S3
**Phase diagrams in the** 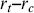 **parameter space for cis-inhibition with different cooperativities in ligand inhibition.** (A–C) Phase diagrams in the 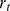
–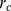 parameter space for (A) no cooperativity (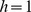), (B) low (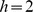) and (C) high (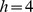) cooperativity. (A) In the absence of cooperativity (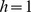), a minimal amount of cis-interactions is required to create a pattern for any 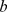 value, being consistent with Sprinzak *et al*. (2010) [25]. (B) At low (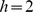) cooperativity, cis-interactions enable spontaneous patterning. (C) At high cooperativity (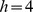) cis-interactions can promote the bistable regions. In all panels, 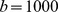 and 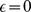. Color codes and line types as in Fig. S2.(TIFF)

Figure S4
**Cis-interactions in the cis-inhibition regime make cells worse receivers of inhibition.** (A) Threshold for lateral inhibition 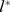 (Eq. 19 with 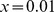 and 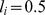) as a function of the trans-interactions strength 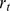 for 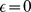. Results for different cis-interactions strengths are depicted: 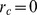 (solid line), 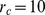 (gray dashed line) and 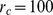 (dotted-dashed gray line). The vertical line is a guide to the eye for a particular trans-interactions strength value, to better appreciate the rise of 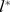 due to cis-interactions strength. (B–C) Contour lines for different 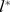 values are depicted across the 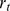
–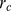 parameter space for (B) 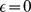 and (C) 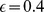. Lines are depicted for 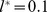 (long-dashed), 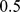, 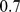, 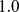 (short-dashed). As a guide to the eye, the spontaneous pattern formation regions (enclosed by blue lines) and the regions where the pattern is a stable solution of the dynamics (enclosed by green dashed lines) are depicted. Other parameter values are as in Fig. S3C.(TIFF)

Figure S5
**Cis-inhibiting interactions increase the ratio of high-ligand expressing cells at** 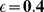 **.** Simulation results showing patterns of ligand levels from precursors with large initial variability between them for different cis-interactions strengths (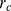). Grayscale is used to denote the ligand level (black for the highest ligand activity, 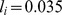, and white for no ligand activity, 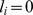). Other parameter values are 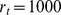, 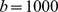, 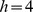 and 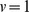.(TIFF)

Figure S6
**Representation of periodic patterns composed of two cell types on a regular hexagonal array.** (A) Salt-and-pepper patterns with the periodicity of the fastest growing mode (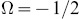, see LSA in Text S1). The P and I patterns have the same periodicity but 33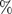 and 66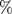 of cells, respectively, are high-ligand expressing cells (black). (B) Patterns with the periodicities of the secondary fastest growing modes (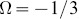, see LSA in Text S1). P2 and I2 are salt-and-pepper patterns too with 25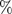 and 75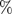 of high-ligand expressing cells respectively. The pattern of stripes (S) has 50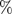 of cells with high ligand levels. On the right of each row of patterns, two groups of 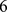 cells neighboring a central cell illustrate how many neighboring cells are like the central one and how many are different. Each group has a different cell type on the center (cell type 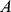 in violet and cell type 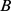 in green). Notice that cell types 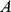 and 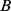 are defined by the 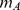 and 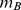 values (Methods) and not by their ligand level. These illustrations facilitate the computation of 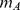 and 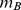 values of Eqs. 16–18 for each pattern.(TIFF)

Figure S7
**Cis-inhibiting interactions enable the salt-and-pepper pattern with 66** 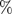 **of cells highly expressing the ligand.** (A) Phase diagram showing where patterns (green) P and (red) I (as defined in Fig. S6) are each a stable solution of the dynamics to small perturbations. Green dashed and blue lines as in Fig. S3C. (B) Bifurcation diagrams for each cell type, 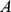 and 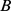, for 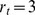. The periodic solutions are shown in blue. The homogeneous solution is shown in gray. Solid (dashed) lines correspond to linearly stable (unstable) states. At low cis-interactions strengths, the stable branches correspond to P (light blue) and at higher cis-interactions strengths to I (dark blue). Note that there is a large parameter region in which both patterns are stable. Solutions for patterns were found by solving Eqs. 16–18 with 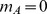 and 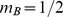. Stability of solutions was evaluated through numerical simulations of the dynamics (Text S1). Parameter values: 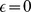, 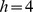, 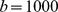 and 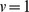.(TIFF)

Figure S8
**Cis-inhibiting interactions enable the salt-and-pepper pattern with 75** 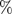 **of cells highly expressing the ligand.** (A) Phase diagram showing where patterns (green) P2 and (red) I2 (as defined in Fig. S6) are each a stable solution of the dynamics to small perturbations. Green dashed and blue lines as in Fig. S3C. (B) Bifurcation diagrams for each cell type, 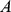 and 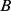, for 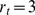. The periodic solutions are shown in blue. The homogeneous solution is shown in gray. Solid (dashed) lines correspond to linearly stable (unstable) states. At low cis-interactions strengths, the stable branches correspond to P2 (light blue) and at higher cis-interactions strengths to I2 (dark blue). Note that there is a large parameter region in which both patterns are stable. Solutions for patterns were found by solving Eqs. 16–18 with 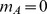 and 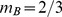. Stability of solutions was evaluated through numerical simulations of the dynamics (Text S1). Parameter values: 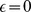, 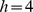, 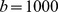 and 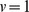.(TIFF)

Figure S9
**Cis-inhibiting interactions enable the stripped pattern with 50** 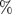 **of cells highly expressing the ligand.** (A) Phase diagram showing where pattern (green) S (as defined in Fig. S6) is a stable solution of the dynamics to small perturbations. Green dashed and blue lines as in Fig. S3C. (B) Bifurcation diagrams for each cell type, 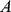 and 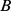, for 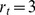. The periodic patterned solution is shown in blue. The homogeneous solution is shown in gray. Solid (dashed) lines correspond to linearly stable (unstable) states. Since type 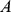 and type 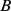 cells are equivalent for this pattern, there is bistability of the stripes solution. In all the parameter region where the stripped pattern is stable there are several patterns (P, I, P2 or P2) that are stable too (Figs. S7, S8). Solutions for patterns were found by solving Eqs. 16-18 with 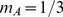 and 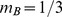. Stability of solutions was evaluated through numerical simulations of the dynamics (Text S1). Parameter values: 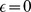, 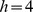, 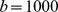 and 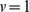.(TIFF)

Figure S10
**Sensitivity to initial conditions occurs at high cis-interactions strengths in the cis-inhibition regime.** (A–C) Stationary patterns of ligand level emerging from precursors with large initial random variability between them. (D–F) Structure function (Text S1) of the patterns in A–C respectively, without the homogeneous mode (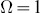). (A,D) The disordered pattern that emerges at high cis-interactions strengths (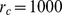), where the homogeneous solution is linearly stable. (B,E) Regular pattern that emerges at lower cis-interactions strengths (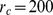), where the homogeneous solution is linearly stable. In these panels we set 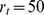. (C,F) Salt-and-pepper periodic pattern that emerges within the spontaneous pattern formation region, for very low cis-interactions strengths (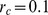 and 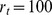). Other parameter values are 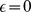, 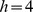, 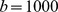 and 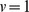.(TIFF)

Figure S11
**The switch between cis-activation and cis-inhibition regulatory roles also occurs in the Complex model.** Stationary Notch signal in cell 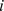, Eq. 11, *versus* the amount of free ligand in the cell, 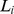, and the primary signaling source for (A–C) the multicellular system (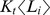 with 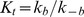) and (D–F) the single cell system (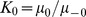) for (A,D) null (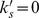), (B,E) slow (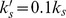 in B, and 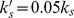 in E) and (C,F) fast 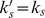 cis-signaling. The value of 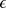 is (A,D) 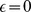, (B) 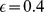, (E) 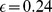 and (C,F) 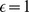. Red lines show the dependence of 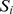 on 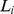 when there is no primary source and when it is maximal on the plot. An increasing function denotes cis-activation, while a decreasing function corresponds to cis-inhibition. A,D (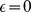) show cis-inhibition; B,E (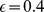 and 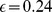) show a switch from cis-activation to cis-inhibition as the primary source increases; D,F (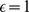) show cis-activation. Other parameter values: 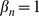 au hr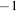, 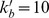 au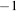 hr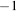, 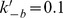 hr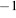, 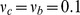 hr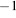, 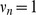 hr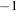 and 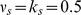 hr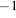 for all panels; 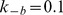 hr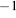 for (A–C) and 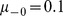 hr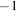 for (D–F). hr refers to hours and au refer to arbitrary concentration units.(TIFF)

Figure S12
**Cis-inhibiting interactions promote higher ratios of high-ligand expressing cells in the Complex model.** Stationary patterns reached by numerical integration of the dynamics for different cis-interactions strengths as measured through the cis-binding rates 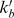 values below the panels (in au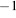 hr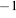 units). Ligand levels are represented in grayscale (black for 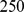 and white for 0). Lower cis-binding affinities (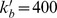) allow high-ligand expressing cells next to each other [25]. Higher cis-affinities drive a gradual increase of the ratio of ligand-positive cells in the tissue. Herein this phenomenology occurs even in the absence of cooperativity (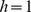). Parameter values are in the cis-inhibition regime: 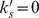, 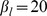 au hr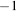, 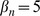 au hr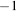, 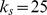 au hr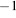, 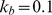 au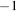 hr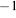, 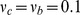 hr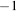, 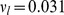 hr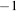, 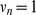 hr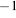, 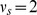 hr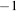, 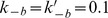 hr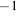, 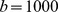 au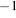, 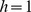. hr refers to hours and au refers to arbitrary concentration units. Precursor cells (initial conditions) were set as 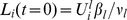 and 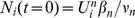 where 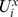 is a uniform random number between 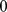 and 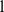, and the remaining variables were set to 0.(TIF)

Figure S13
**Cis-inhibition with a primary Notch signaling source can create cell-autonomous bistability in the Complex model.** Representation of relations S9a–S9b in the phase space of the signal and the ligand levels. Two stable solutions are shown (filled circles) and an unstable solution (empty circle). Stability was evaluated through numerical integration of the dynamics. This bistability occurs even in the absence of any cooperativity (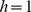). Parameter values in the cis-inhibition regime: 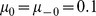 hr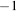, 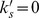, 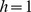, 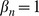 au hr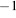, 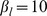 au hr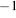, 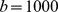 au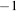, 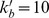 au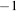 hr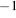, 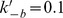 hr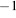, 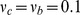 hr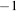, 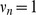 hr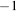, 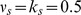 hr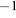 and 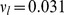 hr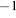. hr refers to hours and au refer to arbitrary concentration units.(TIFF)

Figure S14
**Cell labeling scheme.** Arrays of 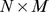 perfect hexagonal cells with the subindex labeling schemes used that number each cell along the array. In (A) one subindex is used, while in (B) we use two subindices. The two main spatial directions of the cellular array are depicted.(TIFF)

Figure S15
**Decomposition of the elements determining the linear stability of the homogeneous state.** Parameter values as in Fig. 3D (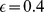, 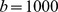 and 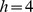). (A) Strength of trans-activation 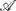 across the 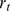
–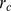 parameter space. (B) Strength of ligand repression 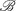. (C) Strength of cis-inhibition (for 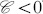) and of cis-activation (for 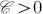). Trans-interactions strengths (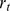) are crucial for determining the cis-role at intermediate cis-signaling efficiencies. Cis-inhibition promotes spontaneous patterning at high cis-interactions strengths (Eq. 14 in Methods). In each panel, color codes are detailed on the color bar.(TIFF)

Figure S16
**Simulation results agree with the spontaneous pattern formation regions predicted from LSA.** Phase diagram in the 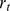
–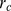 parameter space for 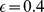 as in Fig. 3D, with the blue triangles indicating the boundaries of the spontaneous pattern formation regions computed from simulation results (Text S1). Other parameter values as in Fig. S3C.(TIFF)

Text S1 **Supplementary text contains more detailed aspects on the models and on the analytical and computational tools being used.**
(PDF)

## References

[1] Artavanis-Tsakonas S, Rand M, Lake R (1999) Notch signaling: cell fate control and signal integration in development. Science 284: 770. 10221902 10.1126/science.284.5415.770

[2] Schwanbeck R, Martini S, Bernoth K, Just U (2011) The notch signaling pathway: molecular basis of cell context dependency. Eur J Cell Biol 90: 572–81. 21126799 10.1016/j.ejcb.2010.10.004

[3] Andersson ER, Sandberg R, Lendahl U (2011) Notch signaling: simplicity in design, versatility in function. Development 138: 3593–612. 21828089 10.1242/dev.063610

[4] Hori K, Sen A, Artavanis-Tsakonas S (2013) Notch signaling at a glance. J Cell Sci 126: 2135–40. 23729744 10.1242/jcs.127308 PMC3672934

[5] Fehon R, Kooh P, Rebay I, Regan C, Xu T, et al (1990) Molecular interactions between the protein products of the neurogenic loci notch and delta, two egf-homologous genes in drosophila. Cell 61: 523–534. 2185893 10.1016/0092-8674(90)90534-l

[6] Kopan R, Ilagan MXG (2009) The canonical notch signaling pathway: unfolding the activation mechanism. Cell 137: 216–33. 19379690 10.1016/j.cell.2009.03.045 PMC2827930

[7] Chitnis A, Henrique D, Lewis J, Ish-Horowicz D, Kintner C (1995) Primary neurogenesis in xenopus embryos regulated by a homologue of the drosophila neurogenic gene delta. Nature 375: 761–766. 7596407 10.1038/375761a0

[8] Haddon C, Smithers L, Schneider-Maunoury S, Coche T, Henrique D, et al (1998) Multiple delta genes and lateral inhibition in zebrafish primary neurogenesis. Development 125: 359. 9425132 10.1242/dev.125.3.359

[9] Kageyama R, Ohtsuka T, Shimojo H, Imayoshi I (2008) Dynamic notch signaling in neural progenitor cells and a revised view of lateral inhibition. Nat Neurosci 11: 1247–1251. 18956012 10.1038/nn.2208

[10] Formosa-Jordan P, Ibañes M, Ares S, Frade JM (2012) Regulation of neuronal differentiation at the neurogenic wavefront. Development 139: 2321–9. 22669822 10.1242/dev.076406

[11] Formosa-Jordan P, Ibañes M, Ares S, Frade JM (2013) Lateral inhibition and neurogenesis: novel aspects in motion. Int J Dev Biol 57: 341–50. 23873365 10.1387/ijdb.120259jf

[12] Heitzler P, Simpson P (1991) The choice of cell fate in the epidermis of drosophila. Cell 64: 1083–1092. 2004417 10.1016/0092-8674(91)90263-x

[13] Collier JR, Monk NA, Maini PK, Lewis JH (1996) Pattern formation by lateral inhibition with feedback: a mathematical model of delta-notch intercellular signalling. J Theor Biol 183: 429–46. 9015458 10.1006/jtbi.1996.0233

[14] Jacobsen TL, Brennan K, Arias AM, Muskavitch MA (1998) Cis-interactions between delta and notch modulate neurogenic signalling in drosophila. Development 125: 4531–40. 9778511 10.1242/dev.125.22.4531

[15] Cordle J, Johnson S, Tay JZY, Roversi P, Wilkin MB, et al (2008) A conserved face of the jagged/serrate dsl domain is involved in notch trans-activation and cis-inhibition. Nat Struct Mol Biol 15: 849–857. 18660822 10.1038/nsmb.1457 PMC2669539

[16] Fiuza UM, Klein T, Arias AM, Hayward P (2010) Mechanisms of ligand-mediated inhibition in notch signaling activity in drosophila. Dev Dyn 239: 798–805. 20063416 10.1002/dvdy.22207

[17] Becam I, Fiuza UM, Arias AM, Milán M (2010) A role of receptor notch in ligand cis-inhibition in drosophila. Curr Biol 20: 554–60. 20226663 10.1016/j.cub.2010.01.058

[18] Fleming RJ, Hori K, Sen A, Filloramo GV, Langer JM, et al (2013) An extracellular region of serrate is essential for ligand-induced cis-inhibition of notch signaling. Development 140: 2039–49. 23571220 10.1242/dev.087916 PMC3631976

[19] Heitzler P, Simpson P (1993) Altered epidermal growth factor-like sequences provide evidence for a role of notch as a receptor in cell fate decisions. Development 117: 1113–1113. 8325237 10.1242/dev.117.3.1113

[20] Sakamoto K, Ohara O, Takagi M, Takeda S, Katsube K (2002) Intracellular cell-autonomous association of notch and its ligands: A novel mechanism of notch signal modification. Dev Biol 241: 313–326. 11784114 10.1006/dbio.2001.0517

[21] Ladi E, Nichols JT, Ge W, Miyamoto A, Yao C, et al (2005) The divergent dsl ligand dll3 does not activate notch signaling but cell autonomously attenuates signaling induced by other dsl ligands. J Cell Biol 170: 983–92. 16144902 10.1083/jcb.200503113 PMC2171428

[22] Glittenberg M, Pitsouli C, Garvey C, Delidakis C, Bray S (2006) Role of conserved intracellular motifs in serrate signalling, cis-inhibition and endocytosis. EMBO J 25: 4697–4706. 17006545 10.1038/sj.emboj.7601337 PMC1618092

[23] Matsuda M, Chitnis AB (2008) Interaction with notch determines endocytosis of specific delta ligands in zebrafish neural tissue. Development 136: 197–206. 19056830 10.1242/dev.027938 PMC2685967

[24] del Álamo D, Schweisguth F (2009) Notch signalling: Receptor cis-inhibition to achieve directionality. Curr Biol 19: R683–R684. 19706274 10.1016/j.cub.2009.07.025

[25] Sprinzak D, Lakhanpal A, Lebon L, Santat LA, Fontes ME, et al (2010) Cis-interactions between notch and delta generate mutually exclusive signalling states. Nature 465: 86–90. 20418862 10.1038/nature08959 PMC2886601

[26] Miller AC, Lyons EL, Herman TG (2009) cis-inhibition of notch by endogenous delta biases the outcome of lateral inhibition. Curr Biol 19: 1378–83. 19631544 10.1016/j.cub.2009.06.042 PMC2761761

[27] del Álamo D, Rouault H, Schweisguth F (2011) Mechanism and significance of cis-inhibition in notch signalling. Curr Biol 21: R40–7. 21215938 10.1016/j.cub.2010.10.034

[28] Yamamoto S, Charng WL, Rana NA, Kakuda S, Jaiswal M, et al (2012) A mutation in egf repeat-8 of notch discriminates between serrate/jagged and delta family ligands. Science 338: 1229–32. 23197537 10.1126/science.1228745 PMC3663443

[29] Meir E, von Dassow G, Munro E, Odell GM (2002) Robustness, exibility, and the role of lateral inhibition in the neurogenic network. Curr Biol 12: 778–86. 12015114 10.1016/s0960-9822(02)00839-4

[30] Hsu CP, Lee PH, Chang CW, Lee CT (2006) Constructing quantitative models from qualitative mutant phenotypes: preferences in selecting sensory organ precursors. Bioinformatics 22: 1375–82. 16522667 10.1093/bioinformatics/btl082

[31] Buceta J, Herranz H, Canela-Xandri O, Reigada R, Sagués F, et al (2007) Robustness and stability of the gene regulatory network involved in dv boundary formation in the drosophila wing. PLoS One 2: e602. 17622347 10.1371/journal.pone.0000602 PMC1904254

[32] Barad O, Rosin D, Hornstein E, Barkai N (2010) Error minimization in lateral inhibition circuits. Sci Signal 3: ra51–ra51. 20606215 10.1126/scisignal.2000857

[33] Lakhanpal A, Sprinzak D, Elowitz MB (2010) Mutual inactivation of notch and delta permits a simple mechanism for lateral inhibition patterning. eprint arXiv 1005: 4301.

[34] Axelrod J (2010) Delivering the lateral inhibition punchline: It's all about the timing. Sci Signal 3: pe38. 20978236 10.1126/scisignal.3145pe38

[35] Sprinzak D, Lakhanpal A, Lebon L, Garcia-Ojalvo J, Elowitz MB (2011) Mutual inactivation of notch receptors and ligands facilitates developmental patterning. PLoS Comput Biol 7: e1002069. 21695234 10.1371/journal.pcbi.1002069 PMC3111533

[36] Wang R, Liu K, Chen L, Aihara K (2011) Neural fate decisions mediated by trans-activation and cis-inhibition in notch signaling. Bioinformatics 27: 3158–3165. 21994231 10.1093/bioinformatics/btr551

[37] Shaya O, Sprinzak D (2011) From notch signaling to fine-grained patterning: Modeling meets experiments. Curr Opin Genet Dev: 1–8. 10.1016/j.gde.2011.07.007 21862316

[38] Barad O, Hornstein E, Barkai N (2011) Robust selection of sensory organ precursors by the notchdelta pathway. Curr Opin Cell Biol 23: 663–7. 21963301 10.1016/j.ceb.2011.09.005

[39] Coumailleau F, Fürthauer M, Knoblich JA, González-Gaitán M (2009) Directional delta and notch trafficking in sara endosomes during asymmetric cell division. Nature 458: 1051–1055. 19295516 10.1038/nature07854

[40] Fürthauer M, González-Gaitán M (2009) Endocytic regulation of notch signalling during development. Traffic 10: 792–802. 19416471 10.1111/j.1600-0854.2009.00914.x

[41] Ghosh S, Paez-Cortez JR, Boppidi K, Vasconcelos M, Roy M, et al (2011) Activation dynamics and signaling properties of notch3 receptor in the developing pulmonary artery. J Biol Chem 286: 22678–22687. 21536678 10.1074/jbc.M111.241224 PMC3121411

[42] Hsieh EH, Lo DD (2012) Jagged1 and notch1 help edit m cell patterning in peyer's patch follicle epithelium. Dev Comp Immunol 37: 306–12. 22504165 10.1016/j.dci.2012.04.003 PMC3374009

[43] Guy CS, Vignali KM, Temirov J, Bettini ML, Overacre AE, et al (2013) Distinct tcr signaling pathways drive proliferation and cytokine production in t cells. Nat Immunol 14: 262–70. 23377202 10.1038/ni.2538 PMC3577985

[44] Childress J, Acar M, Tao C, Halder G (2006) Lethal giant discs, a novel c2-domain protein, restricts notch activation during endocytosis. Curr Biol 16: 2228–2233. 17088062 10.1016/j.cub.2006.09.031 PMC2683616

[45] Miyamoto A, Lau R, Hein PW, Shipley JM, Weinmaster G (2006) Microfibrillar proteins magp-1 and magp-2 induce notch1 extracellular domain dissociation and receptor activation. J Biol Chem 281: 10089–97. 16492672 10.1074/jbc.M600298200

[46] Wilkin M, Tongngok P, Gensch N, Clemence S, Motoki M, et al (2008) Drosophila hops and ap-3 complex genes are required for a deltex-regulated activation of notch in the endosomal trafficking pathway. Dev Cell 15: 762–772. 19000840 10.1016/j.devcel.2008.09.002

[47] Sanders PGT, Muñoz-Descalzo S, Balayo T, Wirtz-Peitz F, Hayward P, et al (2009) Ligandindependent traffic of notch buffers activated armadillo in drosophila. Plos Biol 7: e1000169. 19668359 10.1371/journal.pbio.1000169 PMC2716527

[48] Fortini M, Bilder D (2009) Endocytic regulation of notch signaling. Curr Opin Genet Dev 19: 323–328. 19447603 10.1016/j.gde.2009.04.005 PMC2731830

[49] Fortini M (2009) Notch signaling: the core pathway and its posttranslational regulation. Dev Cell 16: 633–647. 19460341 10.1016/j.devcel.2009.03.010

[50] Cho B, Fischer JA (2011) Ral gtpase promotes asymmetric notch activation in the drosophila eye in response to frizzled/pcp signaling by repressing ligand-independent receptor activation. Development 138: 1349–59. 21350007 10.1242/dev.056002 PMC3114705

[51] Yamada K, Fuwa TJ, Ayukawa T, Tanaka T, Nakamura A, et al (2011) Roles of drosophila deltex in notch receptor endocytic trafficking and activation. Genes Cells 16: 261–72. 21299753 10.1111/j.1365-2443.2011.01488.x

[52] Mukherjee T, Kim WS, Mandal L, Banerjee U (2011) Interaction between notch and hif-alpha in development and survival of drosophila blood cells. Science 332: 1210–3. 21636775 10.1126/science.1199643 PMC4412745

[53] Hori K, Sen A, Kirchhausen T, Artavanis-Tsakonas S (2012) Regulation of ligand-independent notch signal through intracellular trafficking. Commun Integr Biol 5: 374–6. 23060962 10.4161/cib.19995 PMC3460843

[54] Guruharsha KG, Kankel MW, Artavanis-Tsakonas S (2012) The notch signalling system: recent insights into the complexity of a conserved pathway. Nat Rev Genet 13: 654–66. 22868267 10.1038/nrg3272 PMC4369923

[55] Tyson J, Chen K, Novak B (2003) Sniffers, buzzers, toggles and blinkers: dynamics of regulatory and signaling pathways in the cell. Curr Opin Cell Biol 15: 221–231. 12648679 10.1016/s0955-0674(03)00017-6

[56] Formosa-Jordan P, Ibañes M (2009) Diffusible ligand and lateral inhibition dynamics for pattern formation. J Stat Mech 03: 019.

[57] Held W, Mariuzza RA (2011) Cis-trans interactions of cell surface receptors: biological roles and structural basis. Cell Mol Life Sci 68: 3469–78. 21863376 10.1007/s00018-011-0798-z PMC11115084

[58] Owen MR (2002) Waves and propagation failure in discrete space models with nonlinear coupling and feedback. Physica D 173: 59.

[59] Plahte E, Øyehaug L (2007) Pattern-generating travelling waves in a discrete multicellular system with lateral inhibition. Physica D 226: 117–128.

[60] Zhu B (2005) Mechanistic explanation for the unique pharmacologic properties of receptor partial agonists. Biomed Pharmacother 59: 76–89. 15795100 10.1016/j.biopha.2005.01.010

[61] Buchler NE (2005) Nonlinear protein degradation and the function of genetic circuits. P Natl Acad Sci USA 102: 9559–9564. 10.1073/pnas.0409553102 PMC1172234 15972813

[62] Benedito R, Roca C, Sörensen I, Adams S, Gossler A, et al (2009) The notch ligands dll4 and jagged1 have opposing effects on angiogenesis. Cell 137: 1124–1135. 19524514 10.1016/j.cell.2009.03.025

[63] Falix FA, Aronson DC, Lamers WH, Gaemers IC (2012) Possible roles of dlk1 in the notch pathway during development and disease. Biochim Biophys Acta 1822: 988–95. 22353464 10.1016/j.bbadis.2012.02.003

[64] Wang MM (2011) Notch signaling and notch signaling modifiers. Int J Biochem Cell B 43: 1550–62. 10.1016/j.biocel.2011.08.005 PMC3395424 21854867

[65] Digiuni S, Schellmann S, Geier F, Greese B, Pesch M, et al (2008) A competitive complex formation mechanism underlies trichome patterning on arabidopsis leaves. Mol Syst Biol 4: 217. 18766177 10.1038/msb.2008.54 PMC2564731

[66] Kim Y, Coppey M, Grossman R, Ajuria L, Jiménez G, et al (2010) Mapk substrate competition integrates patterning signals in the drosophila embryo. Curr Biol 20: 446–51. 20171100 10.1016/j.cub.2010.01.019 PMC2846708

[67] Kim Y, Andreu MJ, Lim B, Chung K, Terayama M, et al (2011) Gene regulation by mapk substrate competition. Dev Cell 20: 880–7. 21664584 10.1016/j.devcel.2011.05.009 PMC3580161

[68] Cookson NA, Mather WH, Danino T, Mondragón-Palomino O, Williams RJ, et al (2011) Queueing up for enzymatic processing: correlated signaling through coupled degradation. Molecular Systems Biology 7: 561. 22186735 10.1038/msb.2011.94 PMC3737734

[69] Rondelez Y (2012) Competition for catalytic resources alters biological network dynamics. Physical Review Letters 108: 18102. 10.1103/PhysRevLett.108.018102 22304295

[70] Descalzo SM, Rué P, Faunes F, Hayward P, Jakt LM, et al (2013) A competitive protein interaction network buffers oct4-mediated differentiation to promote pluripotency in embryonic stem cells. Mol Syst Biol 9: 694. 24104477 10.1038/msb.2013.49 PMC3817399

[71] Nikolaou N, Watanabe-Asaka T, Gerety S, Distel M, Köster RW, et al (2009) Lunatic fringe promotes the lateral inhibition of neurogenesis. Development (Cambridge, England) 136: 2523–33. 10.1242/dev.034736 PMC2709061 19553285

[72] Panin V, Papayannopoulos V, Wilson R, Irvine K (1997) Fringe modulates notch-ligand interactions. Nature 387: 908–912. 9202123 10.1038/43191

[73] Cohen M, Baum B, Miodownik M (2011) The importance of structured noise in the generation of self-organizing tissue patterns through contact-mediated cell-cell signalling. J R Soc Interface 8: 787–98. 21084342 10.1098/rsif.2010.0488 PMC3104346

[74] Simakov DSA, Pismen LM (2013) Discrete model of periodic pattern formation through a combined autocrine-juxtacrine cell signaling. Phys Biol 10: 046001. 23735823 10.1088/1478-3975/10/4/046001

[75] Stamataki D, Holder M, Hodgetts C, Jeffery R, Nye E, et al (2011) Delta1 expression, cell cycle exit, and commitment to a specific secretory fate coincide within a few hours in the mouse intestinal stem cell system. PLoS One 6: e24484. 21915337 10.1371/journal.pone.0024484 PMC3168508

[76] Tanemura M, Honda H, Yoshida A (1991) Distribution of differentiated cells in a cell sheet under the lateral inhibition rule of differentiation. J Theor Biol 153: 287–300. 1798334 10.1016/s0022-5193(05)80571-5

[77] Parks AL, Huppert SS, Muskavitch MA (1997) The dynamics of neurogenic signalling underlying bristle development in drosophila melanogaster. Mech Develop 63: 61–74. 10.1016/s0925-4773(97)00675-8 9178257

[78] Rauskolb C, Irvine KD (1999) Notch-mediated segmentation and growth control of the drosophila leg. Dev Biol 210: 339–50. 10357895 10.1006/dbio.1999.9273

[79] Stollewerk A, Schoppmeier M, Damen WGM (2003) Involvement of notch and delta genes in spider segmentation. Nature 423: 863–5. 12815430 10.1038/nature01682

[80] Reed R (2004) Evidence for notch-mediated lateral inhibition in organizing buttery wing scales. Dev Genes Evol 214: 43–46. 14618402 10.1007/s00427-003-0366-0

[81] Joshi M, Buchanan KT, Shroff S, Orenic TV (2006) Delta and hairy establish a periodic prepattern that positions sensory bristles in drosophila legs. Dev Biol 293: 64–76. 16542648 10.1016/j.ydbio.2006.01.005

[82] Guantes R, Poyatos JF, Miyano S (2008) Multistable decision switches for exible control of epigenetic differentiation. PLoS Comput Biol 4: e1000235. 19043543 10.1371/journal.pcbi.1000235 PMC2580821

[83] Cheng Z, Liu F, Zhang XP, Wang W (2008) Robustness analysis of cellular memory in an autoactivating positive feedback system. FEBS letters 582: 3776–82. 18930050 10.1016/j.febslet.2008.10.005

[84] Chang HH, Oh PY, Ingber DE, Huang S (2006) Multistable and multistep dynamics in neutrophil differentiation. BMC Cell Biol 7: 11. 16507101 10.1186/1471-2121-7-11 PMC1409771

[85] Lubensky DK, Pennington MW, Shraiman BI, Baker NE (2011) A dynamical model of ommatidial crystal formation. Proc Natl Acad Sci USA 108: 11145–50. 21690337 10.1073/pnas.1015302108 PMC3131319

[86] François P, Hakim V (2005) Core genetic module: the mixed feedback loop. Phys Rev E 72: 031908. 10.1103/PhysRevE.72.031908 16241483

[87] Rouault H, Hakim V (2012) Different cell fates from cell-cell interactions: core architectures of two-cell bistable networks. Biophys J 102: 417–26. 22325263 10.1016/j.bpj.2011.11.4022 PMC3274795

[88] Bu P, Chen KY, Chen JH, Wang L, Walters J, et al (2013) A microrna mir-34a-regulated bimodal switch targets notch in colon cancer stem cells. Cell Stem Cell 12: 602–15. 23642368 10.1016/j.stem.2013.03.002 PMC3646336

[89] Kladko K, Mitkov I, Bishop A (2000) Universal scaling of wave propagation failure in arrays of coupled nonlinear cells. Phys Rev Lett 84: 4505–4508. 10990722 10.1103/PhysRevLett.84.4505

[90] Podgorski G, Bansal M, Flann N (2007) Regular mosaic pattern development: A study of the interplay between lateral inhibition, apoptosis and differential adhesion. Theor Biol Med Model 4: 43. 17974031 10.1186/1742-4682-4-43 PMC2203995

[91] Cross M (2009) Pattern Formation and Dynamics in Nonequilibrium Systems. Cambridge: Cambridge University Press.

[92] Press W, Vetterling W, Teukolsky S, Flannery B (1993) Numerical Recipes in FORTRAN; The Art of Scientific Computing. New York, NY, USA: Cambridge University Press, 2nd edition.

