# Supplementary figures and images for: Competition in Notch Signaling with Cis Enriches Cell Fate Decisions
Source: PLoS One. 2014 Apr 29;9(4):e95744. doi: 10.1371/journal.pone.0095744 (PMC4004554; doi:10.1371/journal.pone.0095744)

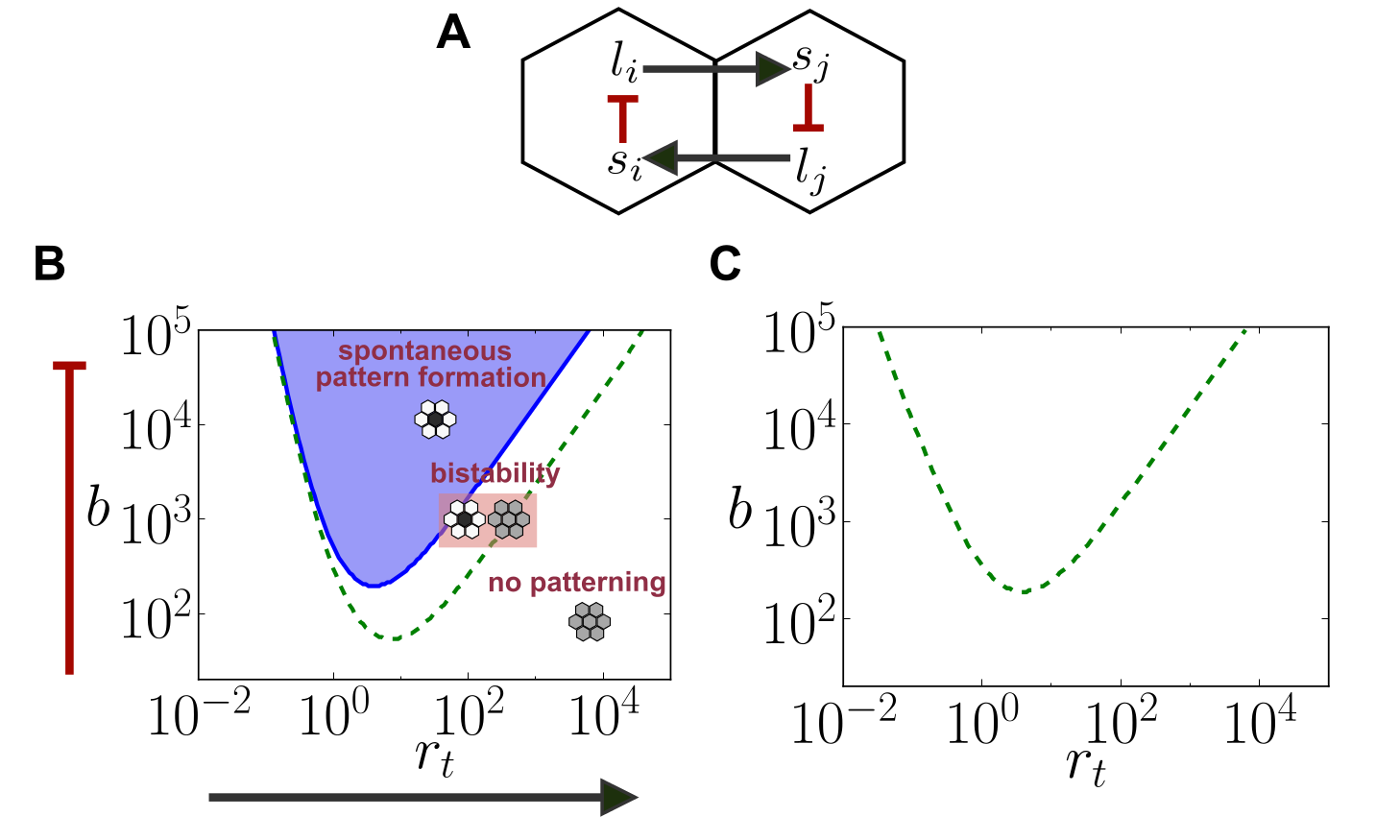

Supplement: Figure S2 — Results in the absence of cis-interactions ( ). (A) Scheme of interactions as in [13] of two cells that inhibit each other through Notch-mediated lateral inhibition. Black (blunt red) arrows denote activation (inhibition). Notice the positive intercellular feedback loop. (B-C) Phase diagrams in the parameter space of ligand inhibition strength and trans-interactions strength for (B) high () and (C) low () cooperativity in ligand inhibition. The blue region in (B) is where the homogeneous state is linearly unstable. This is the region of spontaneous patterning, where the lateral inhibition pattern can arise from the amplification of small differences between precursor cells, as described in [13]. The region above the dashed line is where the pattern solution (with the periodicity shown in Fig. 3A) is an exact stable solution of the dynamics [56]. Above the dashed line and below the blue line in panel B both the homogeneous state and the lateral inhibition pattern are stable solutions of the dynamics (i.e. it is a bistable region). The continuous and dashed lines in (B) have been shown in [10]. Spontaneous patterning does not occur at low cooperativities (). (TIFF) [file pone.0095744.s002.tiff]

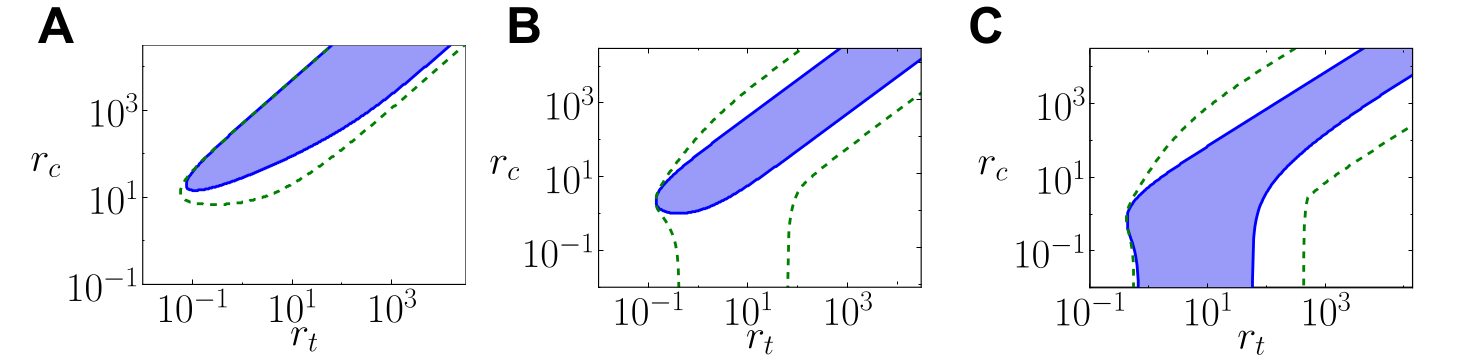

Supplement: Figure S3 — Phase diagrams in the parameter space for cis-inhibition with different cooperativities in ligand inhibition. (A–C) Phase diagrams in the – parameter space for (A) no cooperativity (), (B) low () and (C) high () cooperativity. (A) In the absence of cooperativity (), a minimal amount of cis-interactions is required to create a pattern for any value, being consistent with Sprinzak et al. (2010) [25]. (B) At low () cooperativity, cis-interactions enable spontaneous patterning. (C) At high cooperativity () cis-interactions can promote the bistable regions. In all panels, and . Color codes and line types as in Fig. S2. (TIFF) [file pone.0095744.s003.tiff]

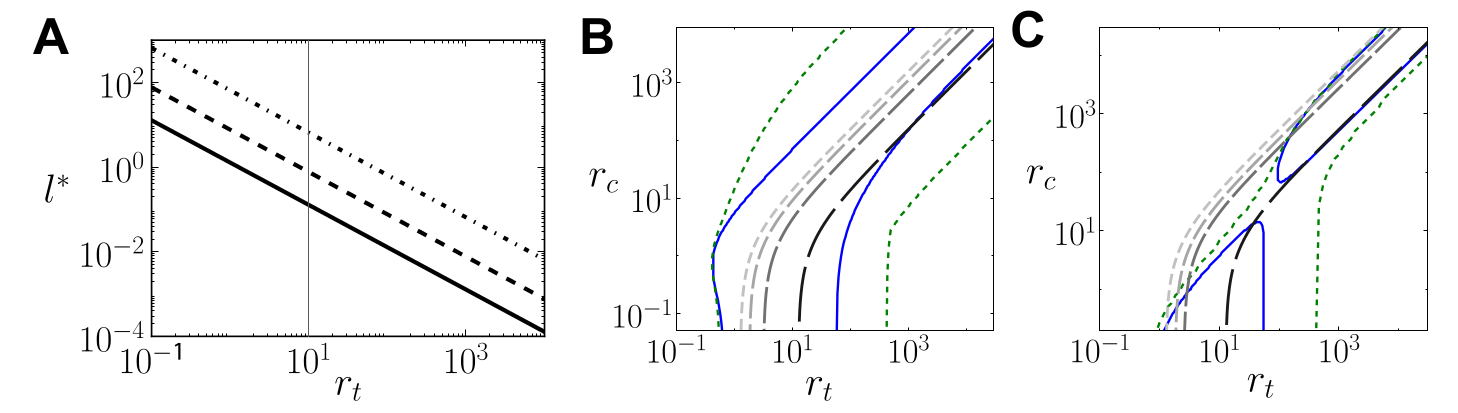

Supplement: Figure S4 — Cis-interactions in the cis-inhibition regime make cells worse receivers of inhibition. (A) Threshold for lateral inhibition (Eq. 19 with and ) as a function of the trans-interactions strength for . Results for different cis-interactions strengths are depicted: (solid line), (gray dashed line) and (dotted-dashed gray line). The vertical line is a guide to the eye for a particular trans-interactions strength value, to better appreciate the rise of due to cis-interactions strength. (B–C) Contour lines for different values are depicted across the – parameter space for (B) and (C) . Lines are depicted for (long-dashed), , , (short-dashed). As a guide to the eye, the spontaneous pattern formation regions (enclosed by blue lines) and the regions where the pattern is a stable solution of the dynamics (enclosed by green dashed lines) are depicted. Other parameter values are as in Fig. S3C. (TIFF) [file pone.0095744.s004.tiff]

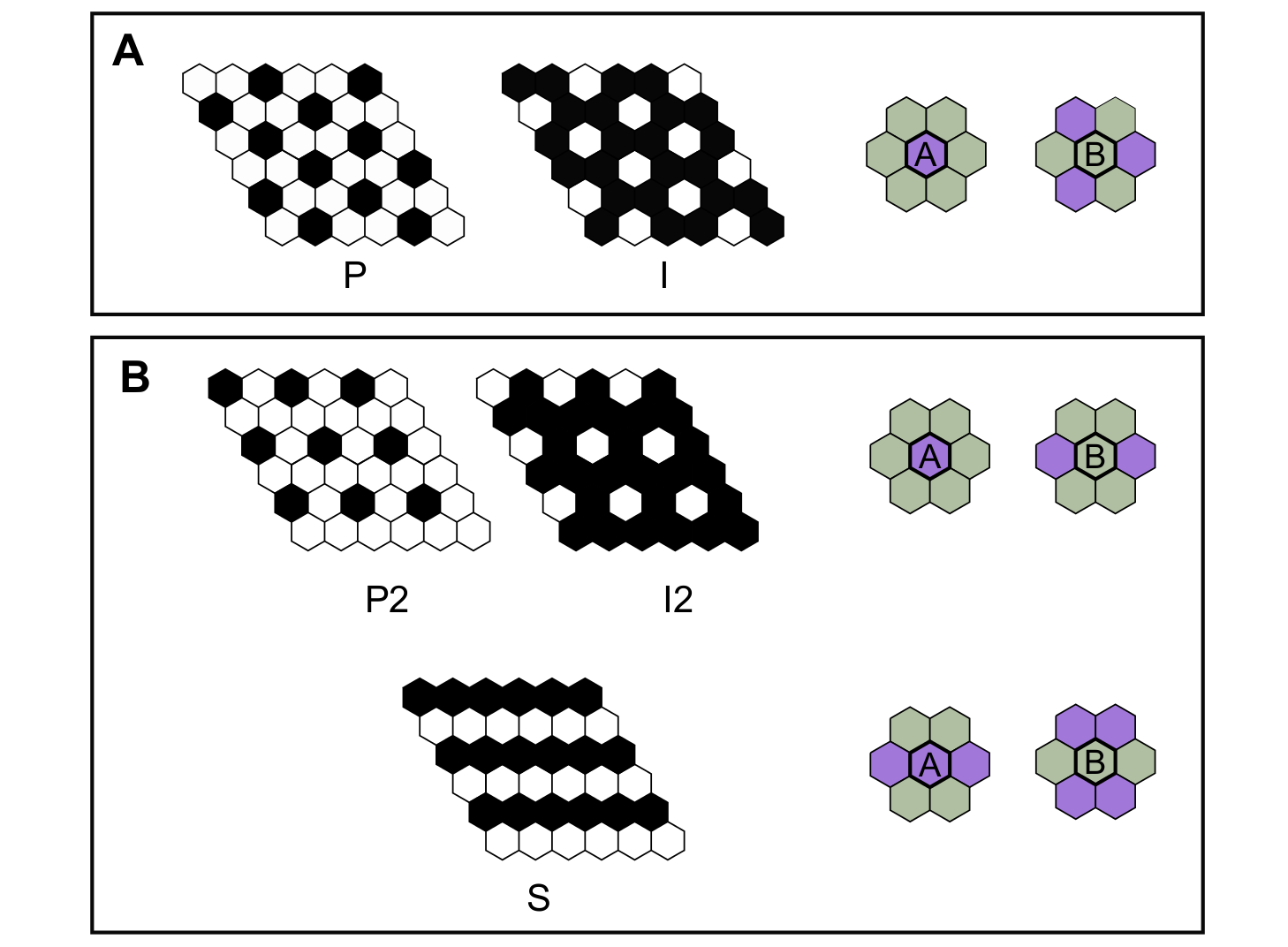

Supplement: Figure S6 — Representation of periodic patterns composed of two cell types on a regular hexagonal array. (A) Salt-and-pepper patterns with the periodicity of the fastest growing mode (, see LSA in Text S1). The P and I patterns have the same periodicity but 33 and 66 of cells, respectively, are high-ligand expressing cells (black). (B) Patterns with the periodicities of the secondary fastest growing modes (, see LSA in Text S1). P2 and I2 are salt-and-pepper patterns too with 25 and 75 of high-ligand expressing cells respectively. The pattern of stripes (S) has 50 of cells with high ligand levels. On the right of each row of patterns, two groups of cells neighboring a central cell illustrate how many neighboring cells are like the central one and how many are different. Each group has a different cell type on the center (cell type in violet and cell type in green). Notice that cell types and are defined by the and values (Methods) and not by their ligand level. These illustrations facilitate the computation of and values of Eqs. 16–18 for each pattern. (TIFF) [file pone.0095744.s006.tiff]

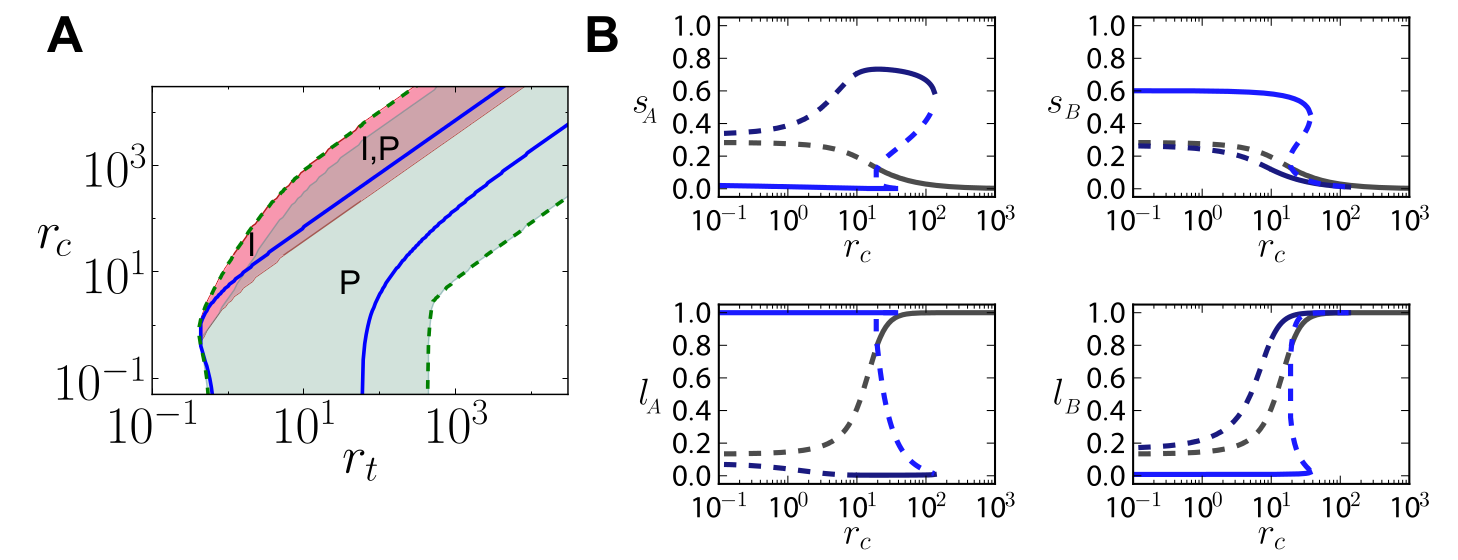

Supplement: Figure S7 — Cis-inhibiting interactions enable the salt-and-pepper pattern with 66 of cells highly expressing the ligand. (A) Phase diagram showing where patterns (green) P and (red) I (as defined in Fig. S6) are each a stable solution of the dynamics to small perturbations. Green dashed and blue lines as in Fig. S3C. (B) Bifurcation diagrams for each cell type, and , for . The periodic solutions are shown in blue. The homogeneous solution is shown in gray. Solid (dashed) lines correspond to linearly stable (unstable) states. At low cis-interactions strengths, the stable branches correspond to P (light blue) and at higher cis-interactions strengths to I (dark blue). Note that there is a large parameter region in which both patterns are stable. Solutions for patterns were found by solving Eqs. 16–18 with and . Stability of solutions was evaluated through numerical simulations of the dynamics (Text S1). Parameter values: , , and . (TIFF) [file pone.0095744.s007.tiff]

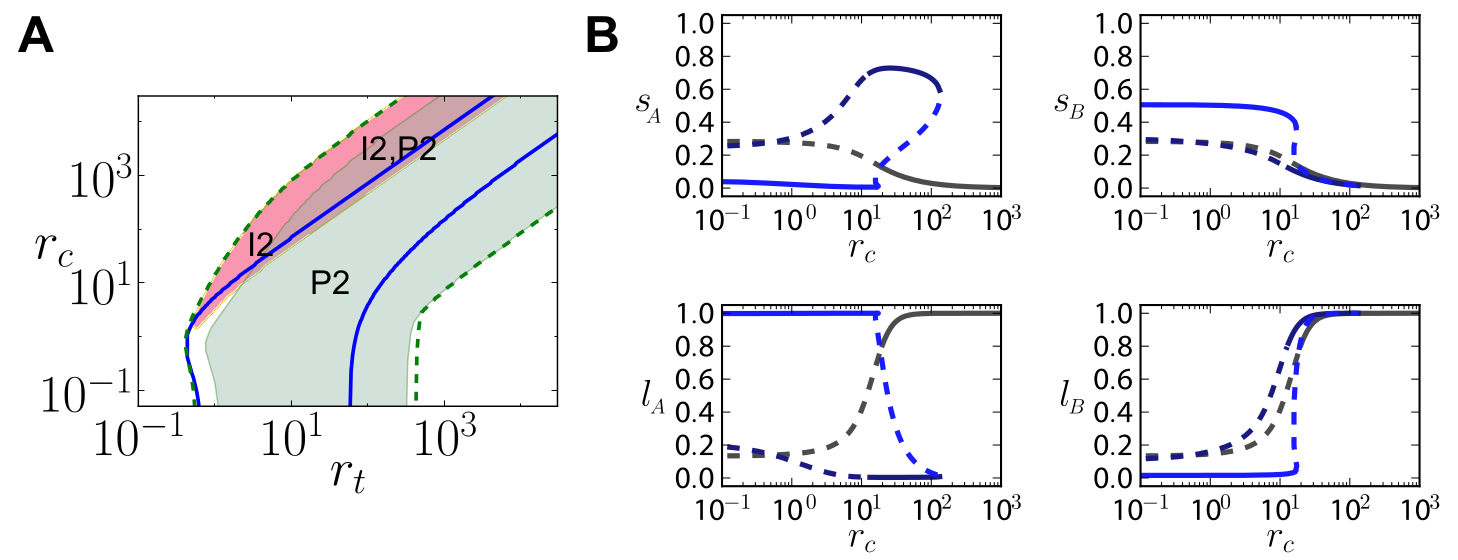

Supplement: Figure S8 — Cis-inhibiting interactions enable the salt-and-pepper pattern with 75 of cells highly expressing the ligand. (A) Phase diagram showing where patterns (green) P2 and (red) I2 (as defined in Fig. S6) are each a stable solution of the dynamics to small perturbations. Green dashed and blue lines as in Fig. S3C. (B) Bifurcation diagrams for each cell type, and , for . The periodic solutions are shown in blue. The homogeneous solution is shown in gray. Solid (dashed) lines correspond to linearly stable (unstable) states. At low cis-interactions strengths, the stable branches correspond to P2 (light blue) and at higher cis-interactions strengths to I2 (dark blue). Note that there is a large parameter region in which both patterns are stable. Solutions for patterns were found by solving Eqs. 16–18 with and . Stability of solutions was evaluated through numerical simulations of the dynamics (Text S1). Parameter values: , , and . (TIFF) [file pone.0095744.s008.tiff]

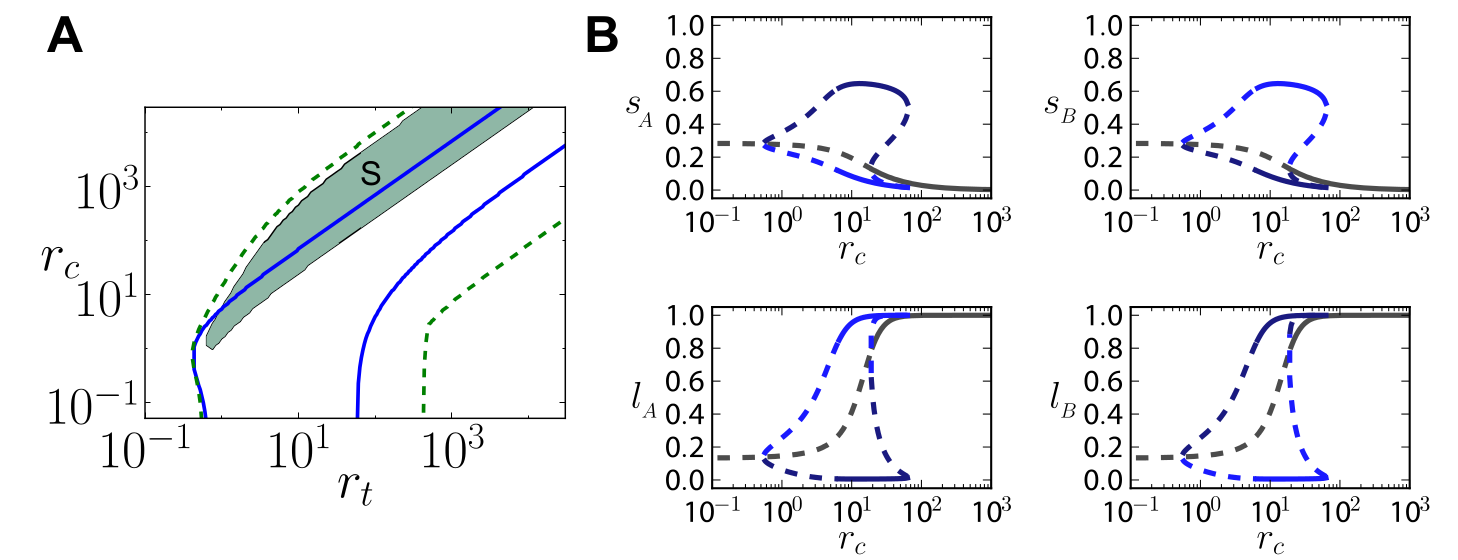

Supplement: Figure S9 — Cis-inhibiting interactions enable the stripped pattern with 50 of cells highly expressing the ligand. (A) Phase diagram showing where pattern (green) S (as defined in Fig. S6) is a stable solution of the dynamics to small perturbations. Green dashed and blue lines as in Fig. S3C. (B) Bifurcation diagrams for each cell type, and , for . The periodic patterned solution is shown in blue. The homogeneous solution is shown in gray. Solid (dashed) lines correspond to linearly stable (unstable) states. Since type and type cells are equivalent for this pattern, there is bistability of the stripes solution. In all the parameter region where the stripped pattern is stable there are several patterns (P, I, P2 or P2) that are stable too (Figs. S7, S8). Solutions for patterns were found by solving Eqs. 16-18 with and . Stability of solutions was evaluated through numerical simulations of the dynamics (Text S1). Parameter values: , , and . (TIFF) [file pone.0095744.s009.tiff]

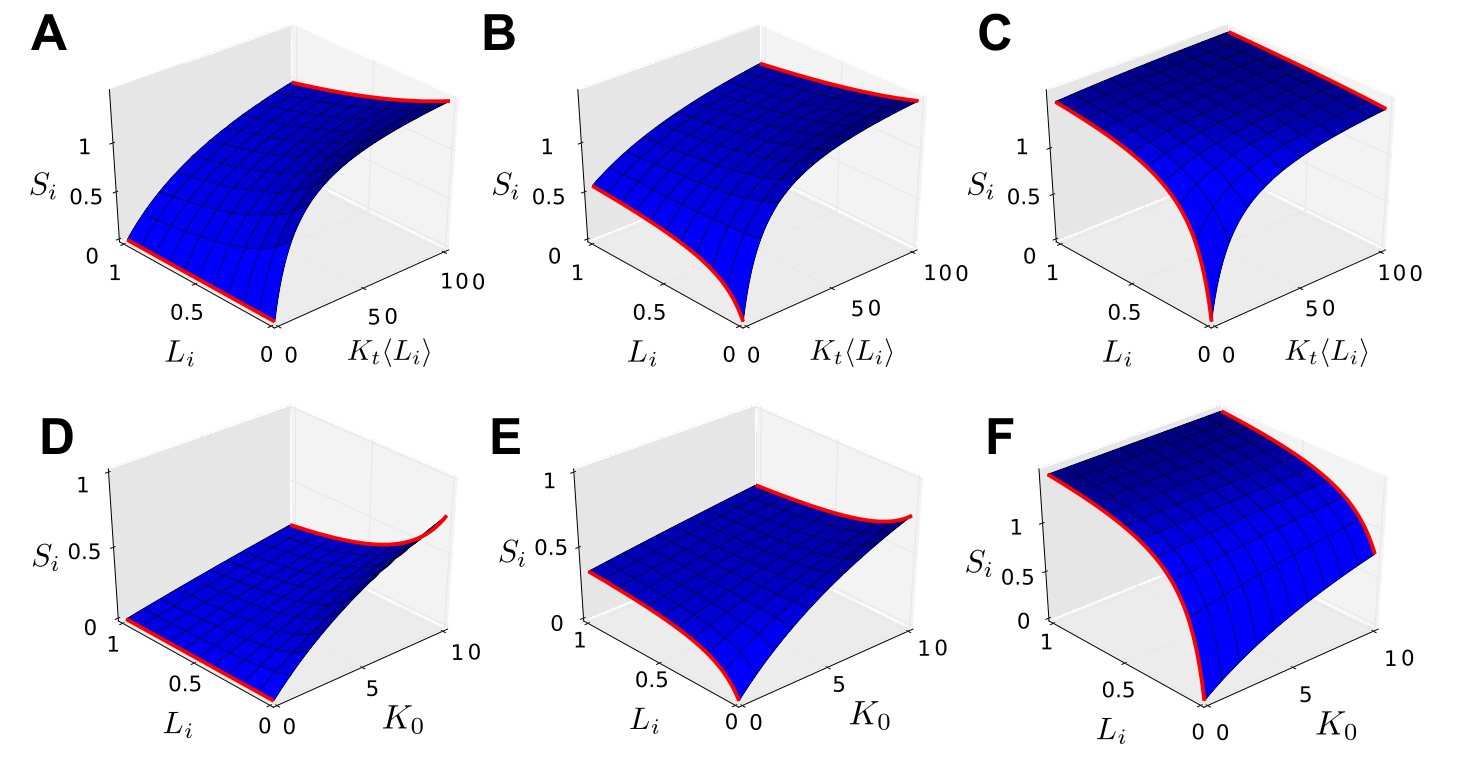

Supplement: Figure S11 — The switch between cis-activation and cis-inhibition regulatory roles also occurs in the Complex model. Stationary Notch signal in cell , Eq. 11, versus the amount of free ligand in the cell, , and the primary signaling source for (A–C) the multicellular system ( with ) and (D–F) the single cell system () for (A,D) null (), (B,E) slow ( in B, and in E) and (C,F) fast cis-signaling. The value of is (A,D) , (B) , (E) and (C,F) . Red lines show the dependence of on when there is no primary source and when it is maximal on the plot. An increasing function denotes cis-activation, while a decreasing function corresponds to cis-inhibition. A,D () show cis-inhibition; B,E ( and ) show a switch from cis-activation to cis-inhibition as the primary source increases; D,F () show cis-activation. Other parameter values: au hr, au hr, hr, hr, hr and hr for all panels; hr for (A–C) and hr for (D–F). hr refers to hours and au refer to arbitrary concentration units. (TIFF) [file pone.0095744.s011.tiff]

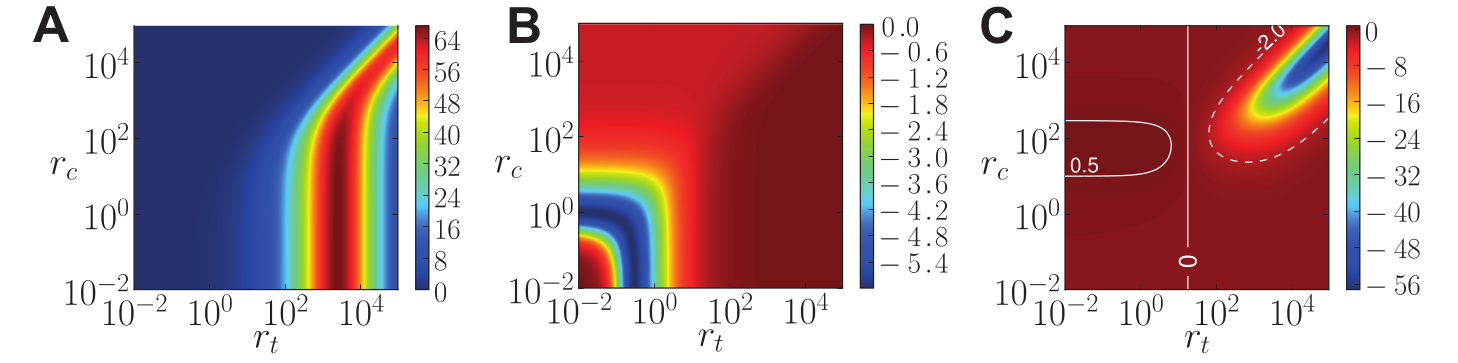

Supplement: Figure S15 — Decomposition of the elements determining the linear stability of the homogeneous state. Parameter values as in Fig. 3D (, and ). (A) Strength of trans-activation across the – parameter space. (B) Strength of ligand repression . (C) Strength of cis-inhibition (for ) and of cis-activation (for ). Trans-interactions strengths () are crucial for determining the cis-role at intermediate cis-signaling efficiencies. Cis-inhibition promotes spontaneous patterning at high cis-interactions strengths (Eq. 14 in Methods). In each panel, color codes are detailed on the color bar. (TIFF) [file pone.0095744.s015.tiff]

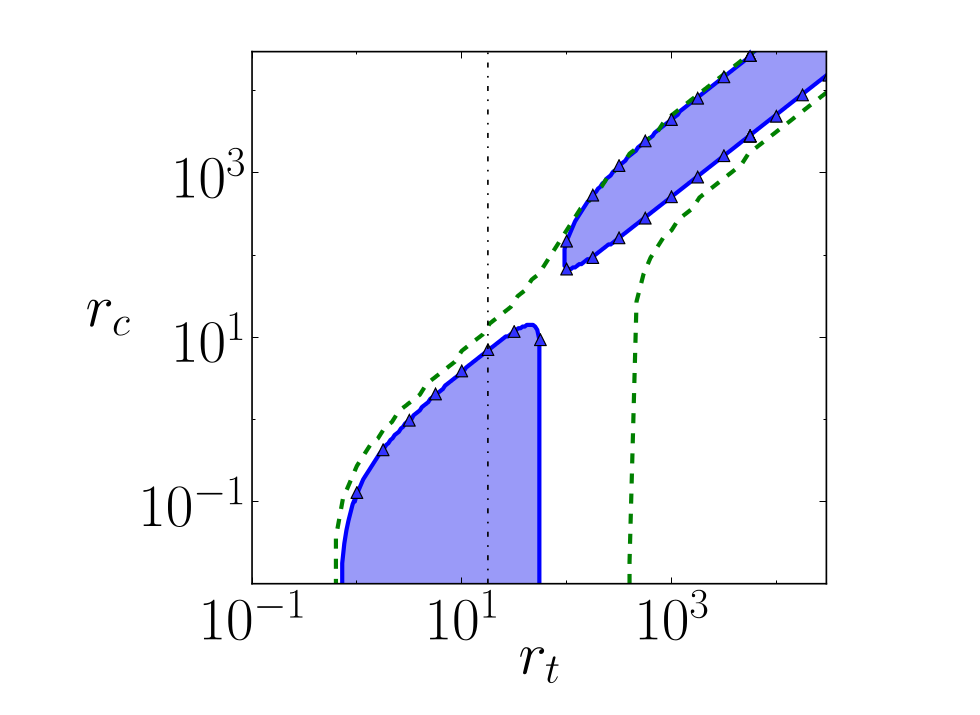

Supplement: Figure S16 — Simulation results agree with the spontaneous pattern formation regions predicted from LSA. Phase diagram in the – parameter space for as in Fig. 3D, with the blue triangles indicating the boundaries of the spontaneous pattern formation regions computed from simulation results (Text S1). Other parameter values as in Fig. S3C. (TIFF) [file pone.0095744.s016.tiff]
