# Supplementary material for: Competition in Notch Signaling with Cis Enriches Cell Fate Decisions
Source: PLoS One. 2014 Apr 29;9(4):e95744. doi: 10.1371/journal.pone.0095744 (PMC4004554; doi:10.1371/journal.pone.0095744)
Supplement: Text S1 — Supplementary text contains more detailed aspects on the models and on the analytical and computational tools being used. (PDF) [file pone.0095744.s017.pdf]

Supplementary Text S1

Pau Formosa-Jordan[1], Marta Ibañes[1,*]
1 Dept. Estructura i Constituents de la Matèria, Facultat de Física, Universitat de Barcelona, Barcelona, Spain
∗ E-mail: mibanes@ub.edu

Contents

1 Complex model formulation and analysis **1**
1.1 Multicellular system 1
1.2 Single cell system 2
1.3 Regulatory role of cis-interactions for the single cell system . 3
1.4 Bistability in the single-cell system 3

2 Spontaneous pattern formation **4**
2.1 Detailed linear stability analysis (LSA) 4
2.2 Numerical integration of the dynamics to check LSA results . 7

3 Numerical evaluation of the stability of periodic pattern solutions **8**

4 Characterization of the periodicity of the pattern through the structure function **9**

1 Complex model formulation and analysis

In this section we use a more detailed model in which we take into account the binding-unbinding dynamics of receptors and ligands, and the proteolytic cleavage of the ligand-receptor forming complex that gives rise to the Notch signal. This model provides a biochemical-based interpretation for some of the parameters used in the phenomenological model. It is also used to verify that our main conclusions extracted with the phenomenological model are not dependent on its approximations.

1.1 Multicellular system

Herein we set that both trans and cis-formed ligand-receptor complexes, C_i and B_i, get proteolytically cleaved, giving rise to the Notch signal (Fig. 1A):

$$N_i + L_j \underset{k_{-b}}{\overset{k_b}{\rightleftharpoons}} C_i \xrightarrow{k_s} S_i \tag{S1a}$$

$$N_i + L_i \underset{k'_{-b}}{\overset{k'_b}{\rightleftharpoons}} B_i \xrightarrow{k'_s} S_i \,. \tag{S1b}$$

We considered as well that these complexes degrade linearly with rates ν_c and ν_b respectively, *i.e*

$$C_i \xrightarrow{\nu_c} \phi, \quad B_i \xrightarrow{\nu_b} \phi\,. \tag{S2}$$

We included basal production and linear degradation for the Notch receptor:

$$\phi \xrightarrow{\beta_n} N_i, \quad N_i \xrightarrow{\nu_n} \phi\,. \tag{S3}$$

For the ligand dynamics we set that ligand production is down-regulated by the signal through the usual Hill function [1]. By considering fast mRNA dynamics, the system of equations for all the dimensional variables takes the following form:

$$\frac{\mathrm{d}L_i}{\mathrm{d}\tau} = -k_b\langle N_i\rangle L_i + k_{-b}\langle C_i\rangle - k_b' N_i L_i + k_{-b}' B_i + \frac{\beta_l}{1+bS_i^h} - -\nu_l L_i \tag{S4a}$$

$$\frac{\mathrm{d}N_i}{\mathrm{d}\tau} = -k_b N_i\langle L_i\rangle - k_b' N_i L_i + k_{-b} C_i + k_{-b}' B_i + \beta_n - \nu_n N_i \tag{S4b}$$

$$\frac{\mathrm{d}C_i}{\mathrm{d}\tau} = k_b N_i\langle L_i\rangle - k_{-b} C_i - k_s C_i - \nu_c C_i \tag{S4c}$$

$$\frac{\mathrm{d}B_i}{\mathrm{d}\tau} = k_b' N_i L_i - k_{-b}' B_i - k_s' B_i - \nu_b B_i \tag{S4d}$$

$$\frac{\mathrm{d}S_i}{\mathrm{d}\tau} = k_s C_i + k_s' B_i - \nu_s S_i\,, \tag{S4e}$$

where the binding and unbinding reactions are explicit in Eqs. S4a-S4d. τ is the dimensional time. We did not perform any additional adiabatic approximation nor nondimensionalization, since our intention is just to check that our main conclusions are also found in a more realistic modeling framework of the Notch signaling pathway.

1.2 Single cell system

We can write an equivalent model to Eqs. S4 for the single cell system (Fig. 1B) by assuming that Notch receptor turns into a modified form C_i in a ligand-independent way, and its cleavage drives signaling:

$$N_i \underset{\mu_{-0}}{\overset{\mu_0}{\rightleftharpoons}} C_i \xrightarrow{k_s} S_i\,. \tag{S5}$$

The corresponding system reads[1]

$$\frac{dL_i}{d\tau} = -k'_b N_i L_i + k'_{-b} B_i + \frac{\beta_l}{1+bS_i^h} - \nu_l L_i \quad \text{(S6a)}$$

$$\frac{dN_i}{d\tau} = -\mu_0 N_i - k'_b N_i L_i + \mu_{-0} C_i + k'_{-b} B_i + \beta_n - \nu_n N_i \quad \text{(S6b)}$$

$$\frac{dC_i}{d\tau} = \mu_0 N_i - \mu_{-0} C_i - k_s C_i - \nu_c C_i \quad \text{(S6c)}$$

$$\frac{dB_i}{d\tau} = k'_b N_i L_i - k'_{-b} B_i - k'_s B_i - \nu_b B_i \quad \text{(S6d)}$$

$$\frac{dS_i}{d\tau} = k_s C_i + k'_s B_i - \nu_s S_i \,. \quad \text{(S6e)}$$

The stationary Notch signal in cell i in terms of the non-dimensional amounts of ligand l_i reads ($dN_i/d\tau = 0$, $dC_i/d\tau = 0$, $dB_i/d\tau = 0$, $dS_i/d\tau = 0$):

$$S_i^{\mathrm{st}} = S_0^{\mathrm{st}} \frac{\mu + \epsilon r_c l_i}{1 + \mu + r_c l_i} \,, \quad \text{(S7)}$$

with $\mu = \frac{\mu_0}{\nu_n} \frac{k_s+\nu_c}{\mu_{-0}+k_s+\nu_c} L_0$ and ϵ, r_c and S_0^{st} being as defined in Methods for the multicellular system (*i.e.* $\epsilon = \frac{k'_s}{k_s} \frac{k_s+\nu_c}{k'_s+\nu_b}$, $r_c = \frac{k'_b}{\nu_n} \frac{k'_s+\nu_b}{k'_{-b}+k'_s+\nu_b} L_0$ and $S_0^{\mathrm{st}} = \frac{k_s \beta_n}{\nu_s(k_s+\nu_c)}$ with L_0 being a characteristic dimensional concentration of ligand).

This model can be easily extended to primary signaling sources that are ligand-dependent yet cell-autonomous.

1.3 Regulatory role of cis-interactions for the single cell system

We quantified the regulatory role of cis-interactions for the single cell system. Following the same procedure as for the multicellular system (described in Methods), and according to Eq. S7, cis-inhibition takes place when

$$\epsilon < \frac{\mu}{1+\mu} \,, \quad \text{(S8)}$$

with μ and ϵ as defined in the previous subsection. Notice the analogy with the multicellular system (Fig. S11).

1.4 Bistability in the single-cell system

We computed the stationary states of the cell-autonomous dynamics (Eqs. S6) by imposing $\frac{dL_i}{d\tau} = 0$, $\frac{dN_i}{d\tau} = 0$, $\frac{dC_i}{d\tau} = 0$, $\frac{dB_i}{d\tau} = 0$ and $\frac{dS_i}{d\tau} = 0$. This

[1]The subindex is kept, but it could be omitted.

results in the following equalities:

$$S_i = S_0^{\rm st}\frac{\mu+\epsilon K_c L_i}{1+\mu+K_c L_i} \tag{S9a}$$

$$S_i = \left(\frac{(\beta_l-\nu_l L_i)(1+\mu+K_c L_i)-\beta_n K_c L_i}{b\,(\nu_l L_i(1+\mu+K_c L_i)+\beta_n K_c L_i)}\right)^{\frac{1}{h}}, \tag{S9b}$$

being $K_c = \frac{r_c}{L_0} = \frac{k'_b}{\nu_n}\frac{k'_s+\nu_b}{k'_{-b}+k'_s+\nu_b}$. Analysis of these equations indicates that more than one stable stationary solution arises for strong cis-interactions (Fig. S13).

2 Spontaneous pattern formation

2.1 Detailed linear stability analysis (LSA)

Linear stability analysis (LSA) over the homogeneous state for the dynamics of the simple model (Eqs. 1-4 and 6 in main text for $\mu = r_t\langle l_i\rangle$) was performed. On one side, LSA indicated in which regions of the parameter space a state of equivalent precursor cells initially exhibiting small variability between them evolves dynamically to a pattern state of several cell types (spontaneous patterning). These regions are delimited by what we call LSA critical lines. On the other side, LSA made a prediction on which is the expected periodicity of the pattern that arises in these regions (what we call the characteristic length of the pattern or pattern wavelength). Such predicted pattern feature comes from the identification of the mode that has the fastest growing rate when the homogeneous state is linearly unstable. This is called the fastest growing mode[2].

Herein we detail how standard LSA calculations apply to our discrete dynamical equations for the multicellular system defined on a regular hexagonal array with periodic boundary conditions. This standard methodology applied to similar kind of problems can be found in different resources [1, 3–5].

First of all we found the homogeneous stationary solution (l_0, s_0) by imposing $l_i = l_j = l_0$, $s_i = s_j = s_0$ $\forall\ i, j$ and $\mathrm{d}l_i/\mathrm{d}t = 0$ and $\mathrm{d}s_i/\mathrm{d}t = 0$ $\forall\ i$. These conditions on Eqs. 1-4 and 6 drive the following algebraic relations

$$l_0 = \frac{1}{1+b s_0^h} \tag{S10a}$$

$$s_0 = \frac{r_t l_0+\epsilon r_c l_0}{1+(r_t+r_c)l_0}, \tag{S10b}$$

[2]The fastest growing mode can be a good predictor of the pattern wavelength when the system is close to the critical line and the pattern formation transition is supercritical [2], *i.e.* the pattern amplitude gradually grows at the critical line. However, even if these conditions do not hold, LSA can be a good starting point to predict the pattern periodicity.

which were solved numerically.

Perturbations $\widetilde{l_i}$ and $\widetilde{s_i}$ to the homogeneous stationary solution were introduced as

$$l_i = l_0 + \widetilde{l_i} \quad \text{(S11a)}$$

$$s_i = s_0 + \widetilde{s_i}\,, \quad \text{(S11b)}$$

where $\widetilde{l_i}$ and $\widetilde{s_i}$ are small. By introducing Eqs. S11 into Eqs. 1-4 and 6, by expanding them as a Taylor series and neglecting second and higher order terms in the perturbations, the linear dynamics of the perturbations were obtained as:

$$\frac{\mathrm{d}}{\mathrm{d}t}\begin{pmatrix} \widetilde{l_i} \\ \widetilde{s_i} \end{pmatrix} \simeq J_0 \begin{pmatrix} \widetilde{l_i} \\ \widetilde{s_i} \end{pmatrix} + J_1 \begin{pmatrix} \sum_{i'} \widetilde{l_{i'}} \\ \sum_{i'} \widetilde{s_{i'}} \end{pmatrix}, \quad \text{(S12)}$$

where isotropy has been taken into account and J_0 and J_1 are the matrices

$$J_0 = \left.\begin{pmatrix} \frac{\partial}{\partial l_i}\frac{\mathrm{d}l_i}{\mathrm{d}t} & \frac{\partial}{\partial s_i}\frac{\mathrm{d}l_i}{\mathrm{d}t} \\ \frac{\partial}{\partial l_i}\frac{\mathrm{d}s_i}{\mathrm{d}t} & \frac{\partial}{\partial s_i}\frac{\mathrm{d}s_i}{\mathrm{d}t} \end{pmatrix}\right|_{l_0,s_0}, \quad J_1 = \left.\begin{pmatrix} \frac{\partial}{\partial l_{i'}}\frac{\mathrm{d}l_i}{\mathrm{d}t} & \frac{\partial}{\partial s_{i'}}\frac{\mathrm{d}l_i}{\mathrm{d}t} \\ \frac{\partial}{\partial l_{i'}}\frac{\mathrm{d}s_i}{\mathrm{d}t} & \frac{\partial}{\partial s_{i'}}\frac{\mathrm{d}s_i}{\mathrm{d}t} \end{pmatrix}\right|_{l_0,s_0}, \quad \text{(S13)}$$

where i' refers to first neighbors to the cell i and all derivatives are computed at the homogeneous state.

In the particular case of a hexagonal array of $N \times M$ cells, the perturbations $\widetilde{l_i}$ and $\widetilde{s_i}$ can be written as

$$\widetilde{l_i} \equiv \widetilde{l_{j,k}} = \sum_{\bar{q}}\sum_{\bar{p}} \sigma^l_{\bar{q},\bar{p}} e^{2\pi \mathrm{i}(\bar{q}j+\bar{p}k)+\alpha_{\bar{q},\bar{p}}t} \quad \text{(S14a)}$$

$$\widetilde{s_i} \equiv \widetilde{s_{j,k}} = \sum_{\bar{q}}\sum_{\bar{p}} \sigma^s_{\bar{q},\bar{p}} e^{2\pi \mathrm{i}(\bar{q}j+\bar{p}k)+\alpha_{\bar{q},\bar{p}}t}\,, \quad \text{(S14b)}$$

where now the two subindexes j, k are used to refer to the spatial position of cell i within the two-dimensional hexagonal cell lattice (see Fig. S14). $\bar{q}$ and $\bar{p}$ are proportional to the wavenumbers along the natural axes in a two-dimensional hexagonal lattice. These wave numbers are defined as $\bar{q} = q/N$, $\bar{p} = p/M$ being $q = 1, ..., N$ and $p = 1, ..., M$, so the first and second sums are performed over $\bar{q} = 1/N, 2/N, ..., 1$ and $\bar{q} = 1/M, 2/M, ..., 1$. Note that the ansatz solutions S14 contain an exponential growth in time of these modes at rate $\alpha_{\bar{q},\bar{p}}$. We referred to the couple of indices $(\bar{q}, \bar{p})$ as a particular Fourier mode. The inverted transforms are

$$\sigma^l_{\bar{q},\bar{p}} = \frac{1}{NM}\sum_j\sum_k \widetilde{l_{j,k}} e^{-2\pi \mathrm{i}(\bar{q}j+\bar{p}k)-\alpha_{\bar{q},\bar{p}}t} \quad \text{(S15a)}$$

$$\sigma^s_{\bar{q},\bar{p}} = \frac{1}{NM}\sum_j\sum_k \widetilde{s_{j,k}} e^{-2\pi \mathrm{i}(\bar{q}j+\bar{p}k)-\alpha_{\bar{q},\bar{p}}t}\,. \quad \text{(S15b)}$$

By introducing Eqs. S14 into the linearized system of differential equations S12, we obtain two coupled algebraic equations per each existing $(\bar{q},\bar{p})$-mode that describe the mode dynamics:

$$\alpha_{\bar{q},\bar{p}}\sigma_{\bar{\mathbf{q}},\bar{\mathbf{p}}} \;=\; \mathrm{L}\sigma_{\bar{\mathbf{q}},\bar{\mathbf{p}}}\,, \tag{S16}$$

being $\sigma_{\bar{\mathbf{q}},\bar{\mathbf{p}}} = \begin{pmatrix} \sigma^{l}_{\bar{q},\bar{p}} \\ \sigma^{s}_{\bar{q},\bar{p}} \end{pmatrix}$, and L is the matrix $\mathrm{L} = \mathrm{J}_0 + \mathrm{J}_1\Omega_{\bar{\mathrm{q}},\bar{\mathrm{p}}}$ where

$$\Omega_{\bar{q},\bar{p}} = \; \tfrac{1}{3}\left\{\cos(2\pi\bar{q}) + \cos(2\pi\bar{p}) + \cos(2\pi(\bar{p}-\bar{q}))\right\} \tag{S17}$$

is the function that takes into account the isotropic spatial coupling terms within the hexagonal lattice. Given that our system is discrete and finite, $\Omega_{\bar{q},\bar{p}}$ takes discrete values within the interval $[-0.5, 1]$.

By diagonalizing the L matrix, the eigenvalues $\alpha_{\bar{q},\bar{p}}$ can be obtained. The eigenvalue $\alpha_{\bar{q},\bar{p}}$ gives the growth rate of the mode $(\bar{q},\bar{p})$. When all modes decrease over time (*i.e.* its real part is negative, so $Re(\alpha_{\bar{q},\bar{p}}) < 0 \;\forall\; (\bar{q},\bar{p})$), the homogeneous state is linearly stable. In contrast, the homogeneous state is linearly unstable when a single mode (or multiple modes) is able to grow exponentially with time (*i.e.* $Re(\alpha_{\bar{q},\bar{p}}) > 0$ for a mode $(\bar{q},\bar{p})$). Since the expression of L depends only on the modes through function $\Omega_{\bar{q},\bar{p}}$, a single growth rate $\alpha(\Omega)$ can be defined for all modes $(\bar{q},\bar{p})$ with the same value Ω of the function $\Omega_{\bar{q},\bar{p}}$ ($\Omega_{\bar{q},\bar{p}} = \Omega$).

For our system, the $\mathrm{L} = \mathrm{J}_0 + \mathrm{J}_1\Omega_{\bar{\mathrm{q}},\bar{\mathrm{p}}}$ matrix reads:

$$\begin{pmatrix} v\mathscr{B} & -v \\ -1 & (\mathscr{C} + \mathscr{A}\Omega_{\bar{q},\bar{p}}) \end{pmatrix}, \tag{S18}$$

being $\mathscr{A}$, $\mathscr{B}$ and $\mathscr{C}$ the following partial derivatives evaluated at the homogeneous stationary solution:

$$\mathscr{B} = \frac{1}{v}\frac{\partial}{\partial s_i}\frac{\mathrm{d}l_i}{\mathrm{d}t}\bigg|_{s_0,l_0} \;=\; -\frac{h s_0^{h-1} b}{(1+b s_0^h)^2} \tag{S19}$$

$$\mathscr{C} = \frac{\partial}{\partial l_i}\frac{\mathrm{d}s_i}{\mathrm{d}t}\bigg|_{s_0,l_0} \;=\; \frac{r_c(\epsilon + r_t l_0(\epsilon-1))}{(1+l_0(r_t+r_c))^2} \tag{S20}$$

$$\mathscr{A} = \omega\,\frac{\partial}{\partial l_{i'}}\frac{\mathrm{d}s_i}{\mathrm{d}t}\bigg|_{s_0,l_0} \;=\; \frac{r_t(1+r_c l_0(1-\epsilon))}{(1+l_0(r_t+r_c))^2}\,, \tag{S21}$$

where $l_{i'}$ is the ligand level in a neighboring cell to cell i. By diagonalizing this L matrix, the eigenvalues $\alpha(\Omega)$ that were obtained are:

$$\alpha(\Omega)^{\pm} = \frac{1}{2}\left\{-(1+v) \pm \left((1+v)^2 - 4v(1-\mathscr{B}(\mathscr{C}+\mathscr{A}\Omega))\right)^{1/2}\right\}. \tag{S22}$$

As indicated before, the homogeneous state is linearly unstable when there is at least one mode that grows exponentially with time, *i.e.* $Re(\alpha(\Omega)^{\pm})$ in

Eq. S22 is positive for at least one mode. This will occur when the following relation is fulfilled:

$$1 < \mathscr{B}(\mathscr{C} + \mathscr{A}\Omega)\,. \tag{S23}$$

Given that $\mathscr{B} < 0$ in all the parameter space and $\mathscr{A} > 0$ for $\epsilon \leq 1$, which is the case we are studying, the fastest growing modes are those ones corresponding to $\Omega = -1/2$ so $(\bar{q}, \bar{p}) = (1/3, 2/3)$ and $(2/3, 1/3)$. These are the same fastest growing modes that destabilize the homogeneous state when only trans-interactions are present (there are no cis-interactions) and that drive the lateral inhibition pattern of Fig. 3A. This result indicates that cis-interactions are not expected to change the characteristic wavelength of the emerging pattern, specially in the regions of the parameter space where the model behaves linearly. This is confirmed by our simulations (Fig. 4A).

According to condition S23 and the fastest growing mode $\Omega = -1/2$, the critical line enclosing the spontaneous pattern formation region (*i.e.* where the homogeneous state is linearly unstable) is determined by

$$1 = \mathscr{B}(\mathscr{C} - \mathscr{A}/2)\,, \tag{S24}$$

which was solved numerically and is represented by solid lines in Figs. 3B-D, 4C, 6D, S2B, S3, S4B-C, S7A, S8A, S9A and S16. These lines were checked through numerical integration of the dynamics as detailed in the next section.

2.2 Numerical integration of the dynamics to check LSA results

The regions where spontaneous patterning was occurring, from the amplification of small initial differences between precursor cells, were determined by Eq. S24. We checked some of these regions by performing numerical integration of the dynamics (Eqs. 1-4 and 6) over a grid of parameter point values in the r_t–r_c parameter space as follows:

- Numerical integration of the dynamics was done as described in Methods for a lattice of 30×30 hexagonal cells.
- The initial conditions were set at the homogeneous state s_0, l_0 with small random variability as described in Methods.
- A single simulation was performed for each parameter point value.
- To evaluate whether at each parameter point the pattern was formed, we computed at the steady state a global function (order parameter)

η_s which is zero for the homogeneous solution and for the x-species it is defined as [5]:

$$\eta_x = \frac{1}{NM}\sum_i \left| x_i - \left(\frac{1}{\omega}\sum_{j\in nn(i)} x_j\right)\right| , \quad \text{(S25)}$$

where the first sum in Eq. S25 is performed over the whole $N \times M$ cells in the tissue, and the second sum is performed only over the ω nearest neighbor cells to cell i.

- By using our grid of parameter point values, we evaluated at which r_t–r_c parameter values a change from $\eta_s \leq 10^{-5}$ to $\eta_s > 10^{-5}$ occurred. This change indicated the transition to spontaneous patterning. We averaged every two subsequent r_t values in our grid for each constant r_c in which this change in η_s was detected.

Fig. S16 exemplifies the agreement of the simulations with the lines delimiting the spontaneous formation region.

3 Numerical evaluation of the stability of periodic pattern solutions

As described in Methods, by solving Eqs. 16-18 we obtained whether a specific periodic pattern composed of two cell types was a solution of the dynamics defined by Eqs. 1-4 on a perfect hexagonal lattice. This method gave also the exact periodic solution. In other words, it gave the levels of each variable s and l for each cell type (A and B).

We evaluated the stability of this exact solution to small perturbations through numerical integration of the dynamics given by Eqs. 1-4 as described as follows:

- Numerical integration of the dynamics was done as described in Methods until reaching the stationary state. This integration was performed on perfect hexagonal arrays of 3×3 cells for the P and I patterns, and of 6×6 cells for the P2, I2 and S patterns (see Fig. S6 for a description of each of these patterns).
- Initial conditions were set to be the exact pattern solution being found, with a small random variability added as described in Methods.
- For each point of the parameter space being analyzed, ten simulations were performed, which differed in the random numbers of the initial conditions.

- We defined a distance of the stationary state reached by the numerical integration of the dynamics to the exact pattern solution, $\Delta\eta$. For each point in the parameter space, the exact pattern solution was taken as stable when $\Delta\eta < 10^{-5}$ for each of the ten simulations performed in such parameter space point.

The distance $\Delta\eta$ was defined as follows:

$$\Delta\eta \equiv \left|(\eta_l - \eta_l^{th}) + (\eta_s - \eta_s^{th})\right| , \tag{S26}$$

being η_x as defined in Eq. S25 for variable x and being η_x^{th} the function η_x evaluated for the exact periodic solution with cell types A and B:

$$\eta_x^{th} = q_A \eta_{x_A}^{th} + q_B \eta_{x_B}^{th} , \tag{S27}$$

being $q_{A,B}$ the cell fraction adopting the A, B-type and where

$$\eta_{x_{A,B}}^{th} = (1 - m_{A,B})|x_A - x_B| \tag{S28}$$

with $m_{A,B}$ as defined in Methods.

4 Characterization of the periodicity of the pattern through the structure function

We made use of the structure function [6] to study the periodicity and randomness in patterning when cis-interactions are present. This function provides the amplitude spectrum of the spatial modes participating in a pattern.

Provided that LSA indicated that all the spatial modes $(\bar{q}, \bar{p})$ with the same value Ω of function $\Omega_{\bar{q},\bar{p}}$ (Eq. S17) grow at the same rate, we computed the structure function of the pattern formed by variable x (*e.g.* the ligand) as a function of Ω by doing the average over all the Ω corresponding modes [5]:

$$\mathbb{S}(\Omega) = \sum_{(\bar{q},\bar{p})\in\Omega} \frac{1}{C(\Omega)} \mathbb{S}(\bar{q}, \bar{p}) , \tag{S29}$$

with $C(\Omega_{\bar{q},\bar{p}})$ being the number of $\bar{q}$,$\bar{p}$ modes that have the same Ω value and

$$\mathbb{S}(\bar{q}, \bar{p}) = \frac{1}{MN} \sum_{j,k} \sum_{j',k'} x_{j,k} x_{j',k'} e^{-2\pi \mathrm{i}(\bar{q}(j-j')+\bar{p}(k-k'))}, \tag{S30}$$

where we used the notation $\sum_{j,k} = \sum_{j=1}^{N} \sum_{k=1}^{M}$ for a lattice of $M \times N$ cells. Herein we used as in LSA two indexes to label the spatial position (j, k) of cell i. For having a better illustration of the contribution of the non-homogeneous modes in the studied patterns, we did not represent the homogeneous mode ($\Omega = 1$) of the structure function in Fig. S10.

References

[1] Collier JR, Monk NA, Maini PK, Lewis JH (1996) Pattern formation by lateral inhibition with feedback: a mathematical model of delta-notch intercellular signalling. J Theor Biol 183: 429–46.

[2] Cross M (2009) Pattern Formation and Dynamics in Nonequilibrium Systems. Cambridge: Cambridge University Press.

[3] Turing A (1952) The chemical basis of morphogenesis. B Math Biol 52: 153–197.

[4] Webb SD, Owen MR (2004) Oscillations and patterns in spatially discrete models for developmental intercellular signalling. J Math Biol 48: 444–76.

[5] Formosa-Jordan P, Ibañes M (2009) Diffusible ligand and lateral inhibition dynamics for pattern formation. J Stat Mech 03: 019.

[6] García-Ojalvo J, Sancho JM (1999) Noise in spatially extended systems. New York: Springer.
